# Probiotics and synbiotics as adjunctive therapies for heart failure: a systematic review with network and pairwise meta-analyses

**DOI:** 10.3389/fnut.2026.1898538

**Published:** 2026-10-01

**Authors:** Qin Liu, Huanxin Xie, Fenwang Lin, Wenrui Huang, Jinxiong Lao

**Affiliations:** 1 The Eighth Clinical Medical College of Guangzhou University of Chinese Medicine, Foshan, China; 2 China United Graduate School of Chinese Medicine, China Academy of Chinese Medical Sciences, China; 3 The Fourth Clinical Medical College, Guangzhou University of Chinese Medicine, Guangzhou, China; 4 Department of Kidney Transplantation, Beijing Tsinghua Changgung Hospital, School of Clinical Medicine, Tsinghua University, Beijing, China; 5 Guangzhou University of Chinese Medicine, Guangzhou, China; 6 Foshan Hospital of Traditional Chinese Medicine, Foshan, China

**Keywords:** gut microbiota, heart failure, network meta-analysis, pairwise meta-analysis, probiotics, randomized controlled trial, synbiotics

## Abstract

**Objective:**

This study aimed to evaluate the use of probiotics and synbiotics as microbiome-directed adjunctive therapies for heart failure (HF), while explicitly determining whether any eligible prebiotic-only trials existed.

**Methods:**

We searched eight English- and Chinese-language databases from inception to 1 May 2026 and conducted expanded sensitivity searches in PubMed, Embase, and Web of Science on 15 July 2026. Random-effects network meta-analysis (NMA) was used only for connected multi-node evidence; two-node evidence was analyzed pairwise. As placebo, usual care, and food comparators were not clinically exchangeable, comparator-stratified sensitivity analyses of direct evidence were conducted using restricted maximum likelihood (REML) and Hartung-Knapp (HK) confidence intervals (CIs).

**Results:**

A total of 12 randomized studies were included in the systematic review (14 reports); 11 contributed to at least one primary or sensitivity quantitative analysis, and 10 contributed to the primary analyses. The revision-stage search audit identified no additional eligible randomized trials and no eligible prebiotic-only trials. Across all the trial populations, 1,021 participants were randomized, 973 of whom were allocated to review-eligible arms. The left ventricular ejection fraction (LVEF) network included 7 studies and 539 participants in the analysis set; however, this network was star-shaped, had no closed loops, and depended on a combined comparator node. The exploratory NMA estimate for multi-strain bacterial probiotics versus the combined comparator was a mean difference (MD) of 2.13 percentage points (95% confidence interval [CI] −0.10 to 4.35). The comparator-stratified sensitivity analysis for usual care yielded an MD of 1.83 percentage points (95% CI −2.33 to 5.98). All synthesized results were assessed as having either some concern or a high risk of bias. The effect estimates were generally imprecise, and the certainty of evidence was low or very low. Adverse event definitions and denominators were inconsistent.

**Conclusion:**

The available evidence is sparse and affected by significant non-transitivity, risk of bias, and imprecision. Network comparisons and rankings should be considered hypothesis-generating; therefore, no probiotic or synbiotic adjunct can be recommended for routine HF management.

**Systematic review registration:**

CRD420261413595. Public URL: https://www.crd.york.ac.uk/PROSPERO/view/CRD420261413595.

## Introduction

Heart failure (HF) remains a major global public health problem, affecting more than 64 million people worldwide and imposing substantial burdens in terms of morbidity, mortality, quality of life, and healthcare costs (1). Contemporary consensus statements define HF as a clinical syndrome caused by structural or functional cardiac abnormalities, with evidence supported by elevated levels of natriuretic peptides or objective signs of congestion (2). Although guideline-directed medical therapy, device therapy, and multidisciplinary care have substantially improved outcomes, residual symptoms, recurrent hospitalizations, and disease progression remain common across HF phenotypes (3, 4). Therefore, safe adjunctive strategies that target mechanisms not fully addressed by standard neurohormonal and metabolic therapies are clinically relevant.

The gut–heart axis has become an increasingly plausible therapeutic target in HF. Venous congestion, intestinal edema, hypoperfusion, and altered intestinal permeability may favor gut dysbiosis and microbial translocation, thereby amplifying systemic inflammation and oxidative stress (5, 6). Microbiota-derived metabolites are also implicated in HF pathobiology. Trimethylamine N-oxide (TMAO), which is generated from dietary choline, betaine, and L-carnitine through gut microbial metabolism, has been associated with adverse outcomes in patients with HF (7). These observations provide a biological rationale for interventions that modulate the intestinal microbiota, epithelial barrier function, microbial metabolites, and inflammatory signaling.

Probiotics, prebiotics, and synbiotics are related but distinct microbiome-directed interventions. Probiotics are live microorganisms that, when consumed in adequate amounts, provide health benefits to the host (8). Prebiotics are substrates that are selectively utilized by host microorganisms and confer health benefits (9). Synbiotics combine live microorganisms with selectively utilized substrates (10). We searched for all three categories, but the eligible randomized evidence consisted only of probiotic and synbiotic interventions; no prebiotic-only trial met the inclusion criteria.

Clinical evidence for HF is emerging but remains fragmented. Existing randomized evidence encompasses yeast probiotics, bacterial multi-strain capsules or tablets, probiotic yogurt, and synbiotic formulations, with substantial variation in HF phenotype, treatment duration, comparator definition, biomarker selection, and trial quality. As microbiome-directed interventions differ in microbial species, strain composition, dose, formulation matrix, and mechanism, their efficacy is unlikely to represent a uniform class effect. Conventional pairwise meta-analyses that pool these interventions under a broad probiotic label may therefore obscure clinically relevant formulation-specific differences.

Network meta-analysis (NMA) can organize connected direct and indirect evidence, but valid comparative inference requires exchangeable comparators and sufficiently similar distributions of effect modifiers (11). We therefore used NMA as an exploratory framework for connecting multi-node outcomes, analyzed two-node outcomes using pairwise meta-analyses, and conducted comparator-stratified sensitivity analyses of direct evidence.

## Methods

### Protocol and reporting

This systematic review and meta-analysis was reported in accordance with the Preferred Reporting Items for Systematic Reviews and Meta-Analyses (PRISMA) 2020 statement and its extension for network meta-analyses (12, 13). The review was registered on the International Prospective Register of Systematic Reviews (PROSPERO) on 2 June 2026 (CRD420261413595). At the time of registration, the formal processes of searching/study identification and screening were reported as started, while data extraction, risk-of-bias assessment, and data synthesis had not yet started. The record listed a review start date of 3 June 2026; therefore, we report the chronology descriptively and do not make an unqualified claim of prospective registration. Analysis amendments and *post hoc* decisions are classified in Supplementary Table S20.1.

### Eligibility criteria

The review question was framed according to the Population, Intervention, Comparator, Outcomes, and Study Design (PICOS) framework: Adults with HF; probiotic, prebiotic, or synbiotic interventions alone or as adjuncts; placebo, usual care, active food comparators, or other eligible microbiome-directed interventions; cardiac function, natriuretic peptides, inflammatory and gut-derived biomarkers, functional status, readmission, mortality, and safety; and randomized controlled trials. Studies involving patients with myocardial infarction without confirmed HF were excluded. No eligible prebiotic-only randomized trial was identified.

### Information sources and search strategy

We searched PubMed/MEDLINE, Embase, Web of Science Core Collection, the Cochrane Central Register of Controlled Trials (CENTRAL), China National Knowledge Infrastructure (CNKI), Wanfang Data, the Chinese Science and Technology Periodical Database (VIP), and the Chinese Biomedical Literature Database (CBM/SinoMed) from inception to 1 May 2026, along with trial registries, reference lists, and relevant reviews. The search strategies covered intervention classes, current and historical organism names, fermented food formulations, and common prebiotic substrates, without requiring randomized design terms at the database level. To test the review’s sensitivity to broader terminology and update the search, expanded searches for PubMed, Embase, and Web of Science were conducted on 15 July 2026. These searches retrieved 260, 843, and 373 records, respectively. In PubMed, 133 records were unique to the expanded terminology relative to the narrower class-term query over the same date range. All incremental PubMed records and all Embase and Web of Science titles were screened; no additional eligible randomized trial was identified. The original PRISMA flow dated 1 May is retained as the primary search flow, and the revision-stage audit is reported separately in Supplementary Appendix 1.

### Study selection and data extraction

Two reviewers independently screened titles, abstracts, and full texts and extracted data using a predesigned format. Multiple reports from the same randomized population were linked and treated as a single study. For dichotomous outcomes, event counts and outcome-specific denominators were extracted. For continuous outcomes, endpoint means, standard deviations, and analysis-set sample sizes were preferentially extracted. We distinguished among (i) randomization across the complete parent trial, (ii) randomization to review-eligible arms, and (iii) participants contributing to each outcome synthesis. In the GutHeart trial, 151 participants were randomized in the complete trial, but only the *Saccharomyces boulardii* (*S. boulardii*; *n* = 52) and standard care (*n* = 51) arms were eligible. The left ventricular ejection fraction (LVEF) analysis set comprised 46 and 45 participants, respectively.

### Intervention node classification

Interventions were classified as *Saccharomyces boulardii* (*S. boulardii*), multi-strain bacterial probiotics, probiotic yogurt containing *Lactobacillus acidophilus* (*L. acidophilus*) La5 and *Bifidobacterium lactis* (*B. lactis*) Bb12, or multi-strain synbiotics. Comparator conditions were classified separately as placebo, usual/standard HF care, ordinary yogurt food control, or dietary management. The exploratory NMA combined placebo and usual/standard care only to preserve connectivity; ordinary yogurt was merged with the combined comparator only in the relevant food comparator network. These combined nodes should not be interpreted as clinically exchangeable. Comparator-stratified pairwise sensitivity analyses retained only repeated direct comparisons within the same comparator class.

### Outcomes

The primary outcome was end-of-treatment left ventricular ejection fraction (LVEF). Secondary outcomes included N-terminal pro-B-type natriuretic peptide (NT-proBNP) or B-type natriuretic peptide (BNP), C-reactive protein (CRP) or high-sensitivity CRP (hs-CRP), tumor necrosis factor alpha (TNF-α), interleukin 6 (IL-6), interleukin 8 (IL-8), trimethylamine N-oxide (TMAO), soluble cluster of differentiation 163 (sCD163), soluble TNF-like weak inducer of apoptosis (sTWEAK), 6-min walk test (6MWT), readmission, mortality, and safety. Activities of daily living (ADL), the EuroQol five-dimension questionnaire and its visual analog scale (EQ-5D/VAS), and the Kansas City Cardiomyopathy Questionnaire (KCCQ) were treated as distinct constructs and summarized narratively rather than combined. Outcomes with disconnected evidence or fewer than two comparable contrasts were not pooled.

Endpoint values were preferred and were not mixed with change scores. In total, 12 study outcome records reported medians and interquartile ranges (IQRs) and were converted to raw-scale means and standard deviations using the method described by Wan et al. (14). These converted values are model-dependent approximations. Ratio-of-means or log-scale synthesis was not feasible because the necessary geometric means or consistently transformable summary data were unavailable across the contributing studies. Sensitivity analyses excluded converted values when at least two comparable contrasts remained; otherwise, the dependence on converted data was stated explicitly or the result was interpreted narratively.

### Risk of bias and certainty of evidence

The Cochrane Risk of Bias 2 tool (RoB 2) was applied to each outcome result (15). All synthesized results were rated as either ‘some concerns’ or ‘high risk’ overall, chiefly because of allocation concealment, deviations from intended interventions, missing data, and selective reporting. Confidence in Network Meta-Analysis (CINeMA) was used for connected multi-node outcomes (16). Prespecified clinical ranges supported transparent imprecision judgments but were not used to claim efficacy.

### Data synthesis and statistical analysis

Analyses were conducted in R 4.4.2 using netmeta 3.3-1, meta 8.2-1, metafor 4.8-0, readxl 1.4-5, openxlsx 4.2.8, and dplyr 1.2.0. Primary random-effects NMA and pairwise meta-analysis models used DerSimonian–Laird tau-squared estimation and normal-approximation with 95% confidence intervals (CIs). Continuous outcomes were summarized as mean differences (MDs) when scales were common and as standardized mean differences (SMDs) when scales differed; binary outcomes were summarized as risk ratios (RRs). A common heterogeneity variance was assumed across comparisons within each network, and heterogeneity was summarized using the I-squared statistic (I^2^) and tau-squared. No eligible synthesis included more than two eligible arms from the same trial; the ineligible GutHeart rifaximin arm was excluded. For binary outcomes, a continuity correction of 0.5 was applied to all four cells of the 2 x 2 table only for studies with zero events in one treatment arm. Studies with zero events in both arms were excluded from relative-risk estimation.

A *post hoc* broad-class random-effects pairwise meta-analysis combined eligible probiotic and synbiotic interventions into a single active class. As this analysis also combines clinically different interventions and comparators, it is reported only as an exploratory sensitivity analysis in Supplementary Appendix 18 and is not used for the principal conclusions.

### Missing and non-standard data

When the standard deviations were missing, they were calculated from standard errors, confidence intervals, *p*-values, or other available statistics when possible. When outcomes were reported as medians with interquartile ranges or ranges, means and standard deviations were estimated using established methods when the assumptions were clinically plausible (14). Figure-only data were retained for future digitalization but were not forced into the main analysis when numeric extraction was unreliable.

### Sensitivity, subgroup, and meta-regression analyses

Sensitivity analyses evaluated the effects of excluding converted values, high-risk results, theses, open-label trials, and the small Yu 2021 HF subgroup. To address comparator heterogeneity and small-study uncertainty, repeated direct comparisons within the same comparator class were reanalyzed using restricted maximum likelihood (REML) tau-squared estimation and Hartung-Knapp (HK) confidence intervals, with the six-case *ad hoc* correction proposed by the Institute for Quality and Efficiency in Health Care (IQWiG6). Meta-regression was not performed because the largest outcome contained only seven contrasts, and region, duration, intervention, and comparator were strongly confounded. Funnel plots and formal small-study tests were not produced because every outcome had fewer than 10 contrasts.

## Results

### Literature selection

The original database searches through 1 May 2026 identified 377 records: PubMed/MEDLINE 119, Embase 97, Web of Science 125, CENTRAL 11, CNKI 7, Wanfang Data 3, VIP 11, and CBM/SinoMed 4. After the removal of 133 duplicates, 244 records were screened. Of these, 38 full-text reports were assessed, and 24 were excluded, leaving 12 randomized studies reported in 14 publications for inclusion in the systematic review (Figure 1). In total, 11 studies contributed to at least one primary or sensitivity quantitative analysis, and 10 contributed to primary analyses. Karim 2022 remained qualitative-only because no eligible endpoint could be extracted, while Yu 2021 was retained only for sensitivity and narrative analyses. The 15 July revision-stage sensitivity searches retrieved 260 records from PubMed, 843 from Embase, and 373 from the Web of Science. The 133 PubMed records unique to the broader terminology comprised 44 animal or preclinical reports, 44 observational or other non-randomized human studies, 30 reviews, protocols, or other non-primary reports, and 15 reports involving the wrong population, condition, or intervention. Complete title screening of Embase and Web of Science likewise identified only already included or companion reports, protocols or registry records, reviews, non-randomized studies, or preclinical studies. No additional eligible randomized trials or eligible prebiotic-only trials were found.

**Figure 1:**
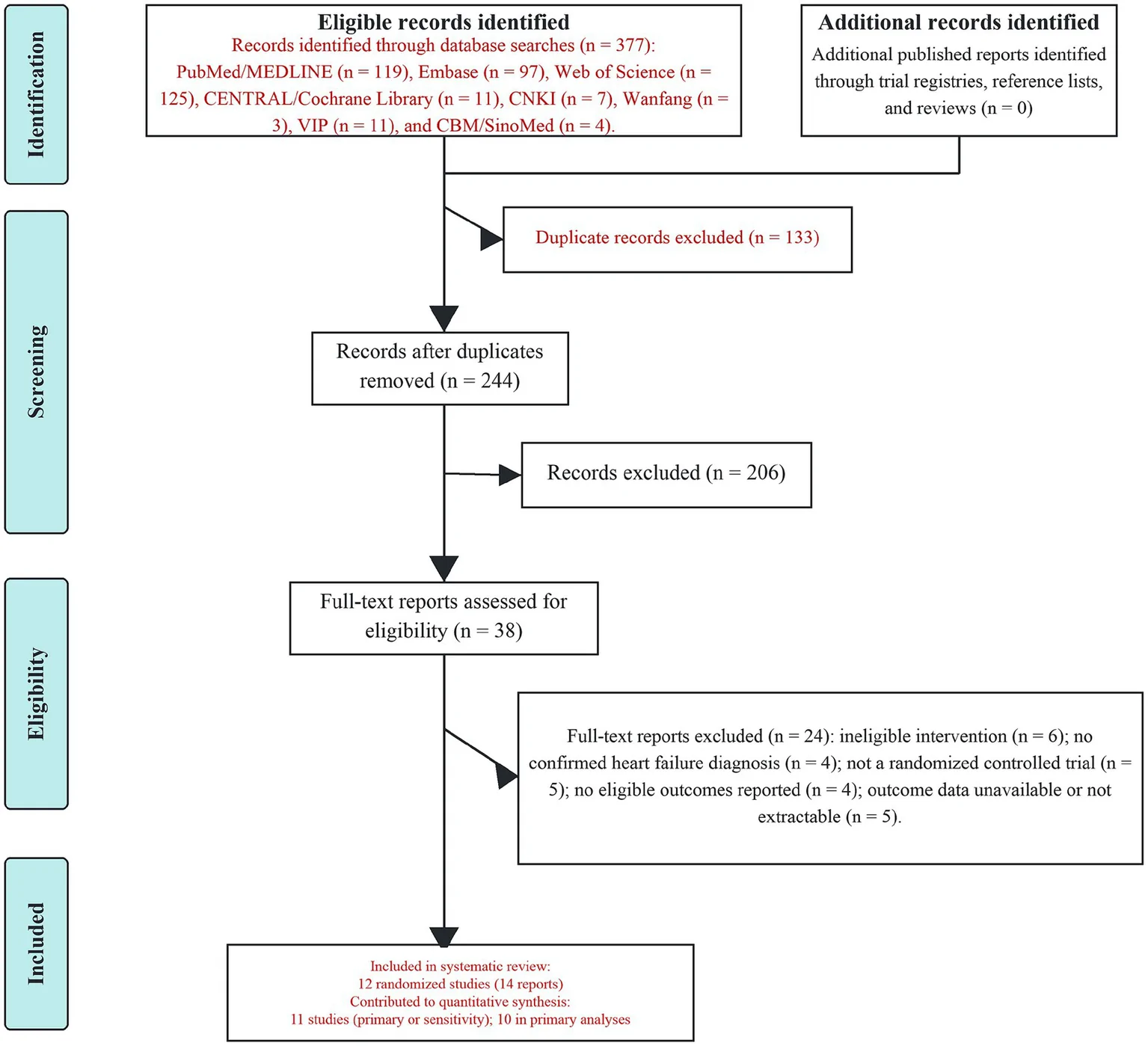
PRISMA flow diagram for the original search through 1 May 2026. In total, 12 randomized studies (14 reports) were included in the systematic review; 11 contributed to at least one primary or sensitivity quantitative analysis, and 10 contributed to primary analyses. Karim 2022 was qualitative-only, while Yu 2021 was included only for sensitivity and narrative analyses. The 15 July 2026 expanded search found no additional eligible randomized trial (Supplementary Appendix 1). CBM/SinoMed, Chinese Biomedical Literature Database; CENTRAL, Cochrane Central Register of Controlled Trials; CNKI, China National Knowledge Infrastructure; MEDLINE, Medical Literature Analysis and Retrieval System Online; PRISMA, Preferred Reporting Items for Systematic Reviews and Meta-Analyses; VIP, Chinese Science and Technology Periodical Database.

### Study characteristics

The systematic review comprised 12 randomized studies reported in 14 articles; 11 studies contributed to at least one primary or sensitivity quantitative analysis, and 10 contributed to primary analyses. The complete trial populations included 1,021 randomized participants; 973 were assigned to review-eligible arms after excluding 48 participants from the GutHeart rifaximin arm. The LVEF synthesis included 539 analysis-set participants across seven studies. Eligible interventions were *S. boulardii*, multi-strain bacterial probiotics, probiotic yogurt, and multi-strain synbiotics; no prebiotic-only trial was identified. Comparator conditions and analysis-set denominators are detailed in Supplementary Table S14.1 (Table 1).

**Table 1:** Basic characteristics of the included randomized studies.

Study	Country/HF phenotype	Design	*N* (I/C)	Intervention node	Comparator/duration	Key outcomes
Costanza 2015 (25)	Brazil; chronic HF with reduced LVEF	Double-blind placebo-controlled pilot RCT	20 randomized; 8/8 analyzed	*S. boulardii*	Placebo + standard HF therapy; 3 months	LVEF; hsCRP; safety
Awoyemi 2021 (26)	Norway; HFrEF	Open-label, randomized, blinded-endpoint, three-arm trial	151 randomized in the full trial; 52/51 randomized to eligible arms; 46/45 in the LVEF analysis set	*S. boulardii*	Standard care; 3 months	LVEF; readmission
Karim 2022 (27)	Pakistan; male CHF, LVEF ≤40%	Double-blind, placebo-controlled RCT	108 randomized; 44/48	Multi-strain bacterial probiotic	Placebo; 12 weeks	CRP; sarcopenia/Wnt signaling biomarkers
Pourrajab 2020/2022 (28, 29)	Iran; CHF, LVEF ≤40%, NYHA I-III	Triple-blind, controlled RCT	90 randomized; 39/39 analyzed	Probiotic yogurt (La5/Bb12)	Ordinary yogurt; 10 weeks	NT-proBNP; oxidative/inflammatory biomarkers
Matin 2022/2024 (30, 31)	Iran; CHF	Triple-blind, placebo-controlled RCT	90 randomized; 41/39 analyzed	Multi-strain synbiotic	Placebo; 10 weeks	NT-proBNP; hsCRP; sCD163/sTWEAK
Ding 2021 (32)	China; chronic HF	Randomized controlled trial	100 randomized; 50/50	Multi-strain bacterial probiotic	Conventional therapy; 12 weeks	TMAO; inflammation; LVEF; 6MWT; ADL
Yu 2021 (33)	China; CHD with a very small HF subgroup	Small randomized subgroup	10 randomized; 5/5	Multi-strain bacterial probiotic	Dietary management; 6 weeks	TNF-α; IL-6; IL-8; CRP
Li 2024 (34)	China; chronic HF	Randomized controlled trial	112 randomized; 56/56	Multi-strain bacterial probiotic	ACEI/ARB + standard therapy; 4 months	LVEF; TMAO; inflammatory markers; readmission; mortality
Chen 2023 (35)	China; HF	Randomized controlled trial	100 randomized; 50/50 for binary outcomes	Multi-strain bacterial probiotic	Conventional HF treatment; 3 months	NT-proBNP; LVEF; TMAO; readmission; mortality
Zhang 2017 (36)	China; older adults with CHF, NYHA II-III, LVEF ≤45%	Single-blind, placebo-controlled RCT	50 randomized; 25/25	Multi-strain bacterial probiotic	Placebo + standard therapy; 4 months	LVEF; EQ-5D; VAS
Xu 2024 (37)	China; chronic HF	Randomized, placebo-controlled thesis	100 randomized; 49/48 completers	Multi-strain bacterial probiotic	Glucose placebo + standard HF therapy; 60 days	NT-proBNP; inflammatory markers; GI reactions
Zhou 2024 (38)	China; older adults with HFpEF	Open-label randomized thesis	90 randomized; 45/45	Multi-strain bacterial probiotic	Usual HFpEF therapy; 12 weeks	KCCQ; 6MWT; LVEF; NT-proBNP; safety; mortality

This table provides a concise main-text summary. Detailed strain, dose, formulation, comparator type, adherence/product verification reporting, and classification notes are provided in Supplementary Appendix Tables S14.1, S14.2. ACEI, angiotensin-converting enzyme inhibitor; ADL, activities of daily living; ARB, angiotensin receptor blocker; CHF, chronic heart failure; CHD, coronary heart disease; CRP, C-reactive protein; Bb12, Bifidobacterium lactis strain Bb12; GI, gastrointestinal; I/C, intervention/control; La5, Lactobacillus acidophilus strain La5; *N*, number of participants; HF, heart failure; HFpEF, heart failure with preserved ejection fraction; HFrEF, heart failure with reduced ejection fraction; hsCRP, high-sensitivity C-reactive protein; IL, interleukin; KCCQ, Kansas City Cardiomyopathy Questionnaire; LVEF, left ventricular ejection fraction; NT-proBNP, N-terminal pro-B-type natriuretic peptide; RCT, randomized controlled trial; TMAO, trimethylamine N-oxide; VAS, visual analog scale; Wnt, Wingless/Integrated signaling pathway; 6MWT, 6-min walk test. No eligible prebiotic-only trial was identified.

### Risk of bias

Outcome-level RoB 2 judgments were either some concern or high risk for every synthesized result; none was judged to be at low risk of bias. Recurrent problems included unclear allocation concealment, open-label deviations, missing outcome data, and selective outcome reporting. Result-specific judgments used in the certainty assessment are provided in Supplementary Table S2.2.

The certainty of evidence was rated as low or very low due to within-study bias, sparse and star-shaped networks, serious concerns regarding comparator and population non-transitivity, heterogeneity, imprecision, and the absence of closed loops. Supplementary Table S17.1 reports the effect estimates, participant counts, and explicit downgrading reasons.

### Network geometry and feasibility

The LVEF network included seven studies, 539 analysis-set participants, three nodes, and two direct comparisons, with no active-versus-active trial and no closed loop. The combined comparator nodes included 265 participants, while the *S. boulardii* and multi-strain bacterial probiotic nodes included 54 and 220 participants, respectively. Other connected networks were similarly sparse and predominantly star-shaped.

Transitivity was seriously threatened. Comparator type, region, HF phenotype, trial design, treatment duration, and intervention composition were unevenly distributed and often aligned with the intervention node. Background guideline-directed therapy, renal function, diabetes, diet, and baseline microbiome characteristics were incompletely reported. Consequently, indirect comparisons between active nodes and all treatment rankings are exploratory rather than confirmatory.

### Primary outcome: left ventricular ejection fraction

In the exploratory combined comparator NMA, multi-strain bacterial probiotics had an LVEF MD of 2.13 percentage points (95% CI −0.10 to 4.35) versus placebo/usual care, while *S. boulardii* had an MD of −0.42 percentage points (95% CI −5.04 to 4.20). In the comparator-stratified direct-evidence sensitivity analysis restricted to bacterial probiotics versus usual/standard care, the REML–Hartung-Knapp estimate was an MD of 1.83 percentage points (95% CI −2.33 to 5.98; four studies; 382 participants; *I*^2^ = 82.4%). These estimates do not establish benefits or comparative superiority (Figure 2).

**Figure 2:**
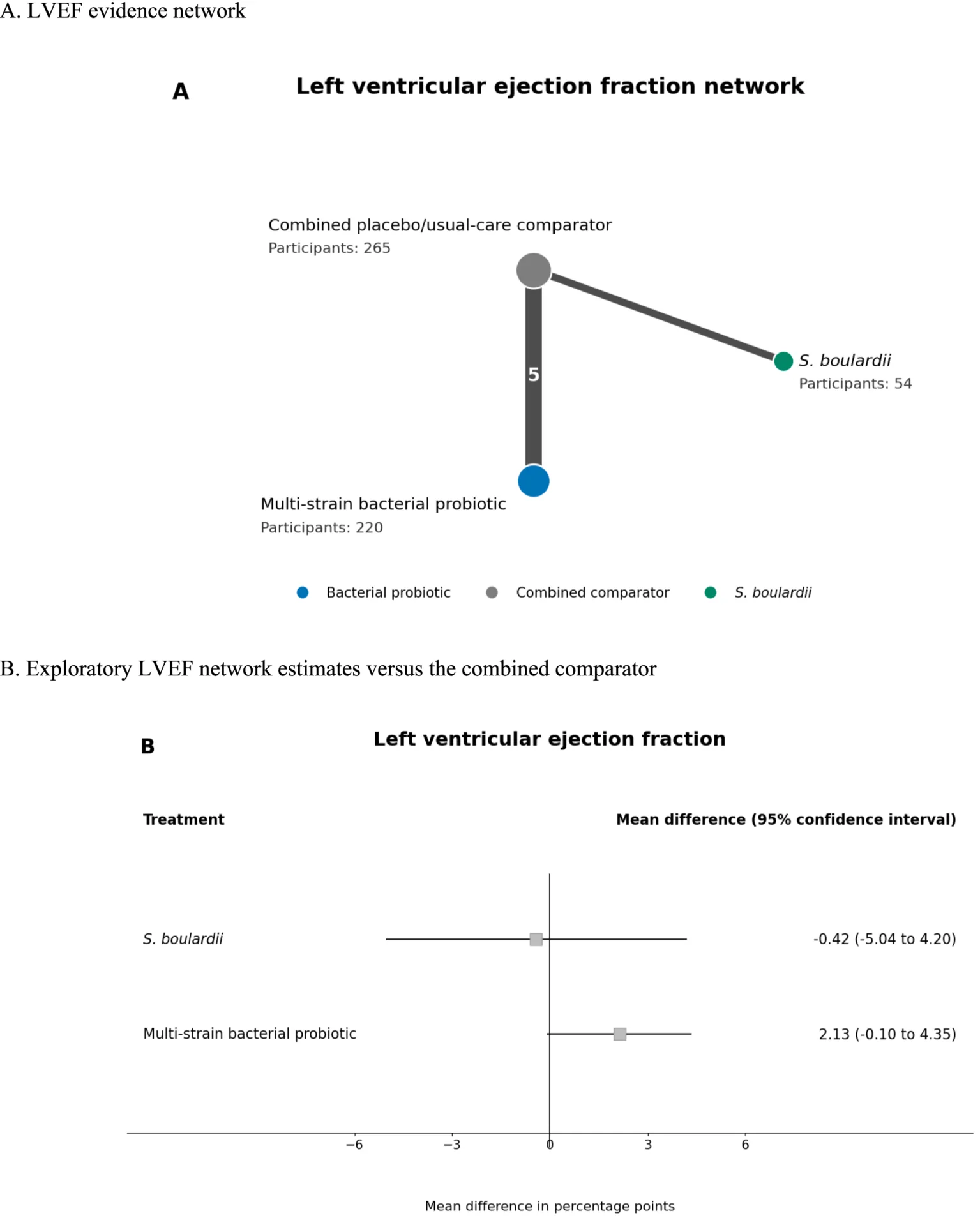
Left ventricular ejection fraction evidence network and exploratory random-effects estimates. *Saccharomyces boulardii* is abbreviated as *S. boulardii* within the panels **(A)** Node area is proportional to the number of analysis-set participants, edge width is proportional to the number of direct trials, and edge labels indicate trial counts. The network contains 539 participants and no closed loop **(B)** Mean differences and 95% confidence intervals are used to compare each active node with the combined placebo/usual care comparator. Positive mean differences favor active intervention; these estimates should not be interpreted as a ranking of active treatments. **(A)** LVEF evidence network. **(B)** Exploratory LVEF network estimates versus the combined comparator.

### Natriuretic peptides and inflammatory outcomes

For NT-proBNP, combined comparator NMA estimates were imprecise for bacterial probiotics (MD −122.99, 95% CI −455.76 to 209.77), synbiotics (MD −175.54, 95% CI −768.25 to 417.17), and probiotic yogurt (MD −12.00, 95% CI −529.05 to 505.05). In the usual care-only REML–Hartung-Knapp sensitivity analysis, the bacterial probiotic estimate was MD −73.50 (95% CI −3524.00 to 3377.00; two studies; 170 participants); both studies relied on median/IQR conversions. No conclusion regarding the NT-proBNP benefit is supported.

For CRP/hs-CRP, the combined comparator NMA estimate for bacterial probiotics was SMD −0.72 (95% CI −1.07 to −0.37), while estimates for *S. boulardii* and synbiotics were imprecise. Each intervention–comparator stratum contained only one study, so a comparator-stratified pooled sensitivity could not be estimated. The apparent signal is therefore vulnerable to confounding by comparator and intervention.

The sCD163 and sTWEAK networks each contained only two studies linked through a combined comparator. All estimates were imprecise, and the absence of active head-to-head evidence precludes reliable comparative interpretation.

The conventional three-study TNF-α analysis, excluding Yu 2021, yielded an MD of −2.35 (95% CI −3.78 to −0.92). Added Yu 2021 to the prespecified sensitivity analysis changed the estimate to an MD of −3.28 (95% CI −4.98 to −1.58). Restricting the analysis to two usual care trials and applying REML–Hartung-Knapp yielded an MD of −3.17 (95% CI −5.00 to −1.33; 212 participants). For TMAO, the corresponding robust estimate was an MD of −0.07 (95% CI −0.16 to 0.02; three studies; 292 participants). IL-6 and IL-8 were each informed by a single comparable study and were not pooled.

### Functional and clinical outcomes

In total, two usual care trials reported 6MWT results. The REML–Hartung-Knapp estimate was an MD of 13.79 m (95% CI −0.56 to 28.15; 190 participants), which was below the approximately 36-m minimal important difference reported for stable chronic HF (17), and the confidence interval included no effect. The ADL, EQ-5D/VAS, and KCCQ results were not pooled because they measure different constructs; the individual study results are narratively summarized in Supplementary Appendix 5.

For readmission, bacterial probiotic trials versus usual care reported 13/106 events versus 31/106; the REML–Hartung-Knapp RR was 0.42 (95% CI 0.01 to 19.09; two studies). The *S. boulardii* trial reported 2/52 versus 3/51 events and was not combined with the bacterial probiotic stratum. For mortality, bacterial probiotic trials reported 3/151 versus 5/151 events; the RR was 0.66 (95% CI 0.03 to 14.01; three studies). These very wide confidence intervals, sparse events, multiple outcomes, and selective outcome availability preclude claims about reduced clinical events. Adverse event definitions and denominators were too inconsistent for pooling; safety evidence was insufficient and not graded, and the absence of demonstrated harm should not be interpreted as reassurance.

The *post hoc* broad-class analysis yielded an LVEF MD of 1.64 (95% CI −0.33 to 3.61), an NT-proBNP MD of −90.66 (95% CI −252.24 to 70.93), and a CRP/hs-CRP SMD of −0.52 (95% CI −0.95 to −0.08). As this analysis collapses non-exchangeable interventions and comparators, it is supplementary and does not support a class-effect conclusion (Table 2).

**Table 2:** Selected exploratory network estimates and comparator-stratified robust pairwise sensitivity analyses.

Outcome	Analysis	Effect measure	Comparison	Estimate (95% CI)
LVEF	Exploratory NMA	MD, percentage points	Bacterial probiotic vs. combined placebo/usual care	2.13 (−0.10, 4.35)
LVEF	Exploratory NMA	MD, percentage points	*S. boulardii* vs. combined placebo/usual care	−0.42 (−5.04, 4.20)
LVEF	Comparator-stratified REML–HK	MD, percentage points	Bacterial probiotic vs. usual/standard care	1.83 (−2.33, 5.98)
NT-proBNP	Exploratory NMA	MD, pg./mL	Bacterial probiotic vs. combined comparator	−122.99 (−455.76, 209.77)
NT-proBNP	Comparator-stratified REML–HK	MD, pg./mL	Bacterial probiotic vs. usual/standard care	−73.50 (−3524.00, 3377.00)
CRP/hs-CRP	Exploratory NMA	SMD	Bacterial probiotic vs. combined comparator	−0.72 (−1.07, −0.37)
TNF-α	Primary DL pairwise	MD	Bacterial probiotic vs. combined comparator	−2.35 (−3.78, −0.92)
TNF-α	Comparator-stratified REML–HK	MD	Bacterial probiotic vs. usual/standard care	−3.17 (−5.00, −1.33)
TMAO	Comparator-stratified REML–HK	MD	Bacterial probiotic vs. usual/standard care	−0.07 (−0.16, 0.02)
6MWT	Comparator-stratified REML–HK	MD, m	Bacterial probiotic vs. usual/standard care	13.79 (−0.56, 28.15)
Readmission	Comparator-stratified REML–HK	RR	Bacterial probiotic vs. usual/standard care	0.42 (0.01, 19.09)
Mortality	Comparator-stratified REML–HK	RR	Bacterial probiotic vs. usual/standard care	0.66 (0.03, 14.01)

All estimates are exploratory. CI, confidence interval; CRP, C-reactive protein; DL, DerSimonian–Laird; hs-CRP, high-sensitivity C-reactive protein; LVEF, left ventricular ejection fraction; MD, mean difference; NMA, network meta-analysis; NT-proBNP, N-terminal pro-B-type natriuretic peptide; REML–HK, restricted maximum likelihood with Hartung-Knapp confidence intervals; RR, risk ratio; SMD, standardized mean difference; TMAO, trimethylamine N-oxide; TNF-α, tumor necrosis factor alpha; 6MWT, 6-min walk test.

### Sensitivity, subgroup, and exploratory analyses

Comparator-stratified direct-evidence sensitivity analyses materially widened uncertainty. For bacterial probiotics versus usual/standard care, robust estimates were an LVEF MD of 1.83 (95% CI −2.33 to 5.98), a 6MWT MD of 13.79 m (−0.56 to 28.15), a TMAO MD of −0.07 (−0.16 to 0.02), a readmission RR of 0.42 (0.01 to 19.09), and a mortality RR of 0.66 (0.03 to 14.01). CRP/hs-CRP had no repeated comparator-specific stratum. These analyses reinforce that the combined comparator NMA signals are not stable enough to support clinical inference.

Formal meta-regression was not performed because the largest synthesis contained seven contrasts, providing inadequate degrees of freedom and severe confounding among region, duration, intervention class, and comparator type. Descriptive subgroup patterns are reported only to show the evidence structure and are not interpreted as effect modification.

Detailed formulation-level and p-score outputs are retained in the supplement solely as descriptive maps of the evidence (40). Most nodes are informed by one study, and the star-shaped networks cannot distinguish treatment effects from study and comparator characteristics; no formulation ranking is used in the conclusion.

### Inconsistency, small-study effects, and evidence certainty

No main network contained a closed loop, so node-splitting and loop-specific inconsistency were not estimable (39). Every outcome had fewer than 10 contrasts; funnel plots and formal tests for small-study effects were therefore not produced. Comparator type, region, HF phenotype, design, and intervention composition remained confounded, contributing to low or very low certainty.

## Discussion

### Principal findings

This review found a small and heterogeneous randomized evidence base. Exploratory combined comparator analyses suggested possible changes in selected biomarkers and outcomes, but comparator-stratified Hartung-Knapp sensitivity analyses were generally imprecise, and the networks contained no closed loops. The evidence therefore serves to identify hypotheses and evidence gaps rather than to establish reliable comparative effects. No eligible prebiotic-only trials were found.

### Interpretation

The gut–heart axis provides a biological rationale for further study, but it does not validate its efficacy. Apparent differences among *S. boulardii*, bacterial probiotics, probiotic yogurt, and synbiotics may reflect differences in region, HF phenotype, comparator, background therapy, duration, or trial quality rather than intervention effects. The biomarker findings, many of which rely on few studies or converted summary data, should not be extrapolated to clinical benefit.

### Biological plausibility and possible mechanisms

Several non-mutually exclusive pathways may connect gut dysbiosis with HF and may also explain why different probiotic and synbiotic formulations should not be assumed to have interchangeable effects. Reduced cardiac output, intestinal hypoperfusion, and venous congestion may disrupt epithelial tight junctions and increase intestinal permeability. This can permit translocation of bacteria or microbial products and amplify innate and adaptive immune activation, systemic inflammation, endothelial dysfunction, and adverse cardiac remodeling. In patients with chronic HF, intestinal permeability and pathogenic bacterial burden have been associated with congestion and inflammation (5). A two-cohort study also identified lower microbial richness and depletion of taxa with butyrate-producing potential, with signals linking these taxa to T-cell activation (18).

Microbial metabolic output provides a second pathway. Trimethylamine N-oxide has been associated with adverse prognosis in HF (7), whereas short-chain fatty acids such as butyrate may support epithelial integrity and restrain inflammatory and fibrotic signaling. In the metabolic syndrome/HF with preserved ejection fraction porcine model highlighted by the Associate Editor, O’Donovan et al. found reduced alpha diversity, enrichment of proinflammatory and secondary bile acid-producing bacteria, and depletion of enteroprotective and short-chain fatty acid-producing bacteria (19). In 1,996 US Hispanic/Latino adults, Luo et al. linked seven gut microbial species and 46 bacteria-associated metabolites—mainly glycerophospholipids, secondary bile acids, and amino acid metabolites—to prevalent left ventricular diastolic dysfunction; 21 of those metabolites were also associated with incident dysfunction (20). These studies support metabolite and barrier pathways but remain experimental or observational and do not prove that modifying the microbiota improves human HF outcomes.

Interventional models add biological plausibility while underscoring formulation specificity. In mice subjected to pressure overload, *Clostridium butyricum*, *Bifidobacterium pseudolongum*, and sodium butyrate attenuated cardiac fibrosis, with combined organisms producing larger effects than either organism alone (21). In a later study using the same porcine cardiometabolic model, a synbiotic containing *Lactobacillus mucosae* plus fermentable fiber improved gut-barrier markers, reduced bacterial translocation and systemic and cardiac inflammation, and attenuated left-sided structural remodeling (22). Taken together, the proposed mechanisms include restoration of barrier integrity, reduced endotoxin exposure, increased short-chain fatty acid availability, altered bile acid and amino acid signaling, and attenuation of inflammatory and profibrotic pathways. However, these effects are likely to depend on strain, viable dose, substrate, diet, background therapy, and host phenotype.

The biomarker outcomes in this review—particularly CRP/hs-CRP, TNF-α, and TMAO—are compatible with some of these pathways, but the trials did not establish mediation. Most studies did not jointly measure microbiome composition, microbial metabolites, intestinal permeability, inflammatory signaling, and cardiac structure, and the clinical estimates remain sparse and uncertain. Future trials should therefore embed longitudinal stool metagenomics, targeted metabolomics, barrier and endotoxemia markers, and prespecified cardiac phenotyping to test whether a defined formulation engages its intended mechanism before inferring clinical efficacy.

### Comparison with previous evidence

Previous pairwise reviews reported possible benefits for selected HF outcomes (23, 24). Our more conservative interpretation follows from linking duplicate reports, separating intervention formulations, distinguishing placebo, usual care, food, and dietary comparators, removing the heterogeneous QoL/ADL pool, and applying comparator-stratified Hartung-Knapp sensitivity analyses. These steps show that nominal signals from broad pooling do not establish a uniform probiotic or synbiotic class effect.

### Clinical and research implications

The findings do not justify routine probiotic or synbiotic use as disease-modifying therapy for HF. Future trials should prespecify one clinically defined formulation and comparator; report strain identity, viable dose, matrix, adherence, diet, antibiotics, and background HF therapy; use validated disease-specific outcomes; and report all clinical events and adverse events with denominators. Direct active-comparator trials are needed before comparative NMA can provide more than exploratory evidence.

### Strengths and limitations

The strengths of the study include outcome-level RoB 2 assessment, explicit participant accounting, a reproducible numerical audit, comparator classification, and robust direct-evidence sensitivity analyses. The limitations of the study include few small trials, no closed loops or active head-to-head comparisons, serious non-transitivity, high heterogeneity, converted skewed biomarker data, heterogeneous outcome instruments, sparse events, multiplicity, selective outcome availability, incomplete safety reporting, and inability to assess small-study effects. P-scores are descriptive only and should not be interpreted as treatment hierarchies.

## Conclusion

Randomized evidence for probiotics and synbiotics in HF remains sparse, heterogeneous, and of low or very low certainty; no eligible prebiotic-only trials were identified. Exploratory network estimates were undermined by the presence of combined comparator nodes, the absence of closed loops, and serious non-transitivity, while comparator-stratified robust sensitivity analyses were mostly imprecise. No microbiome-directed adjunct can currently be recommended for routine HF management.

## Data Availability

The original contributions presented in the study are included in the article/Supplementary material, further inquiries can be directed to the corresponding author.
