# Supplementary material for: Probiotics and synbiotics as adjunctive therapies for heart failure: a systematic review with network and pairwise meta-analyses
Source: Front Nutr. 2026 Oct 1;13:1898538. doi: 10.3389/fnut.2026.1898538 (PMC13668583; doi:10.3389/fnut.2026.1898538)
Supplement: Supplementary file 1 [file Supplementary_file_1.DOCX]

**Supplementary Appendix**

Probiotics and synbiotics as adjunctive therapy for heart failure: supplementary appendix

This appendix reports the audited extraction and analyses. Connected multi-node outcomes are retained as exploratory NMA; two-node outcomes are pairwise; comparator-stratified REML Hartung-Knapp sensitivities are reported separately. No eligible prebiotic-only trial was identified.

# Table of contents

Appendix 1: Search strategy

Appendix 2: Risk of bias of randomized trials

Appendix 3: Evidence geometry, heterogeneity, and inconsistency

Appendix 4: Multi-node network maps and NMA forest plots

Appendix 5: Pairwise meta-analysis forest plots

Appendix 6: Descriptive P-scores for multi-node networks (not used for inference)

Appendix 7: League tables and estimates

Appendix 8: CINeMA certainty framework

Appendix 9: Feasibility of assessing small-study effects

Appendix 10: Sensitivity analyses

Appendix 11: Meta-regression feasibility

Appendix 12: Exploratory detailed formulation-level analyses

Appendix 13: Subgroup analyses

Appendix 14: Intervention/comparator and safety clarifications

Appendix 15: Outcome harmonisation and conversion audit

Appendix 16: Transitivity and primary network accounting

Appendix 17: Summary of findings and CINeMA

Appendix 18: Post hoc broad-class pairwise analysis
Appendix 19: Comparator-stratified robust pairwise sensitivity analyses
Appendix 20: Protocol amendments and analysis decisions

# Appendix 1: Search strategy

Table S1.1. Database-specific expanded strategies. The original systematic searches covered database inception to May 1, 2026. Expanded PubMed, Embase, and Web of Science sensitivity searches were executed on July 15, 2026; the remaining database translations document the reproducible concept mapping but were not rerun at this revision stage.

| **Database** | **Search strategy** |
| --- | --- |
| PubMed/MEDLINE | ("Heart Failure"[Mesh] OR "heart failure"[Title/Abstract] OR "cardiac failure"[Title/Abstract] OR "congestive heart failure"[Title/Abstract] OR HFrEF[Title/Abstract] OR HFpEF[Title/Abstract]) AND ("Probiotics"[Mesh] OR "Prebiotics"[Mesh] OR probiotic*[Title/Abstract] OR prebiotic*[Title/Abstract] OR synbiotic*[Title/Abstract] OR symbiotic*[Title/Abstract] OR microbiota-directed[Title/Abstract] OR microbiome-directed[Title/Abstract] OR Lactobacill*[Title/Abstract] OR Lacticaseibacill*[Title/Abstract] OR Limosilactobacill*[Title/Abstract] OR Bifidobacteri*[Title/Abstract] OR Saccharomyces[Title/Abstract] OR "*Saccharomyces boulardii*"[Title/Abstract] OR "*Clostridium butyricum*"[Title/Abstract] OR yogurt[Title/Abstract] OR yoghurt[Title/Abstract] OR "fermented milk"[Title/Abstract] OR inulin[Title/Abstract] OR oligosaccharide*[Title/Abstract] OR fructooligosaccharide*[Title/Abstract] OR fructo-oligosaccharide*[Title/Abstract] OR galactooligosaccharide*[Title/Abstract] OR galacto-oligosaccharide*[Title/Abstract]) AND ("1900/01/01"[Date - Entrez] : "2026/07/15"[Date - Entrez]) |
| Embase | Set A: ('heart failure'/exp OR 'heart failure':ti,ab OR 'cardiac failure':ti,ab OR 'congestive heart failure':ti,ab OR hfrEF:ti,ab OR hfpef:ti,ab) AND ('probiotic agent'/exp OR probiotic*:ti,ab OR synbiotic*:ti,ab OR lactobacill*:ti,ab OR lacticaseibacillus:ti,ab OR limosilactobacillus:ti,ab OR bifidobacter*:ti,ab OR saccharomyces:ti,ab OR 'clostridium butyricum':ti,ab OR yogurt:ti,ab OR yoghurt:ti,ab OR 'fermented milk':ti,ab OR kefir:ti,ab). Set B: same HF block AND ('prebiotic agent'/exp OR prebiotic*:ti,ab OR inulin:ti,ab OR fructooligosaccharide*:ti,ab OR galactooligosaccharide*:ti,ab OR oligosaccharide*:ti,ab OR 'resistant starch':ti,ab OR 'microbiota directed':ti,ab). Sets A OR B. |
| CENTRAL | ("heart failure" OR "cardiac failure" OR HFrEF OR HFpEF) AND (probiotic* OR prebiotic* OR synbiotic* OR "microbiota-directed" OR Lactobacill* OR Lacticaseibacillus OR Limosilactobacillus OR Bifidobacter* OR Saccharomyces OR "*Clostridium butyricum*" OR yogurt OR yoghurt OR "fermented milk" OR kefir OR inulin OR fructooligosaccharide* OR galactooligosaccharide* OR oligosaccharide* OR "resistant starch") |
| Web of Science | TS=("heart failure" OR "cardiac failure" OR "congestive heart failure" OR HFrEF OR HFpEF) AND TS=(probiotic* OR prebiotic* OR synbiotic* OR "microbiota-directed" OR "microbiota directed" OR Lactobacill* OR Lacticaseibacill* OR Limosilactobacill* OR Bifidobacter* OR Saccharomyces OR "*Saccharomyces boulardii*" OR "*Clostridium butyricum*" OR yogurt OR yoghurt OR "fermented milk" OR kefir OR inulin OR fructooligosaccharide* OR galactooligosaccharide* OR oligosaccharide* OR "resistant starch") |
| CNKI/Wanfang/VIP/CBM | (心力衰竭 OR 心衰 OR 慢性心力衰竭 OR 射血分数降低 OR 射血分数保留) AND (益生菌 OR 益生元 OR 合生元 OR 微生态制剂 OR 双歧杆菌 OR 乳酸杆菌 OR 布拉酵母 OR 酪酸梭菌 OR 芽孢杆菌 OR 活菌 OR 酸奶 OR 发酵乳 OR 菊粉 OR 低聚果糖 OR 低聚半乳糖 OR 低聚糖 OR 抗性淀粉) |
| Trial registries | heart failure AND (probiotic OR prebiotic OR synbiotic OR microbiota OR Lactobacillus OR Lacticaseibacillus OR Bifidobacterium OR Saccharomyces OR yogurt OR fermented milk OR inulin OR oligosaccharide) |

**Table S1.2. Revision-stage expanded-search sensitivity audit (July 15, 2026)**

| **Database** | **Revision-stage records** | **Screening performed** | **Outcome** |
| --- | --- | --- | --- |
| PubMed/MEDLINE | 260 expanded; 127 narrow; 133 incremental | Title/abstract screening of all 133 incremental records | No new eligible randomized trial |
| Embase | 843 (union of probiotic/synbiotic and prebiotic/substrate sets) | Title screening of all 843 records; 38 trial-like titles examined in detail | Included/companion reports only; no new eligible trial |
| Web of Science | 373 records (372 unique titles) | Title screening of all 373 records; 40 trial-signal records examined in detail | Included/companion reports only; no new eligible trial |
| CENTRAL and Chinese databases | No revision-stage rerun | Original May 1 searches retained; expanded concept translations documented in Table S1.1 | Not represented as a rerun |

# Appendix 2: Risk of bias of randomized trials

Risk of bias was evaluated using the Cochrane RoB 2 domains: D1, randomization process; D2, deviations from intended interventions; D3, missing outcome data; D4, measurement of the outcome; D5, selection of the reported result. Trial-level RoB 2 judgments are summarized in Figure S2.1 and Table S2.1.

*Figure S2.1. Risk-of-bias summary across included randomized trials. Bar segments show the number and percentage of trials judged low risk, with some concerns, or high risk in each Cochrane Risk of Bias 2 domain and overall.*

Table S2.1. Trial-level RoB 2 judgments.

| **Study** | **D1** | **D2** | **D3** | **D4** | **D5** | **Overall** | **Rationale** |
| --- | --- | --- | --- | --- | --- | --- | --- |
| Costanza 2015 | Some concerns | Some concerns | Some concerns | Low | Some concerns | Some concerns | Double-blind placebo-controlled pilot, but randomized sample was small and final analyzed numbers were lower than randomized numbers. |
| Awoyemi 2021 | Some concerns | Some concerns | Some concerns | Some concerns | Some concerns | Some concerns | Randomized open-label blinded-endpoint trial; only the probiotic arm was retained for this review. |
| Karim 2022 | Some concerns | Low | Some concerns | Some concerns | Some concerns | Some concerns | Randomized double-blind placebo-controlled trial; attrition from poor compliance and incomplete eligible-outcome reporting. |
| Pourrajab 2020/2022 | Some concerns | Low | Some concerns | Low | Some concerns | Some concerns | Triple-blind controlled trial with linked reports; some outcomes required median/IQR conversion. |
| Matin 2022/2024 | Some concerns | Low | Some concerns | Low | Some concerns | Some concerns | Triple-blind placebo-controlled trial reported across linked publications; selective biomarker availability remains possible. |
| Ding 2021 | Some concerns | Some concerns | Low | Some concerns | Some concerns | Some concerns | Randomization stated, but allocation concealment and blinding were insufficiently described. |
| Yu 2021 | Some concerns | Some concerns | Some concerns | High | High | High | Very small randomized subgroup; subgroup selection and outcome reporting create high concern. |
| Li 2024 | Some concerns | Some concerns | Some concerns | Some concerns | High | High | Randomized trial without clear blinding; internal inconsistencies were noted for some outcome tables. |
| Chen 2023 | Some concerns | Some concerns | High | Some concerns | Some concerns | High | Binary outcomes used randomized denominators, but continuous outcomes used imbalanced completer numbers. |
| Zhang 2017 | Some concerns | Some concerns | Low | Some concerns | Some concerns | Some concerns | Single-blind placebo-controlled trial; several extracted values required table-level verification. |
| Xu 2024 | Some concerns | Some concerns | Some concerns | Some concerns | Some concerns | Some concerns | Placebo-controlled thesis with poor text extraction and several median/IQR biomarker outcomes. |
| Zhou 2024 | Some concerns | High | High | Some concerns | Some concerns | High | Open-label thesis in elderly HFpEF with high attrition; interpreted through sensitivity analyses. |

**Table S2.2. Outcome-level RoB 2 assessments for principal outcomes.**

| **Outcome** | **Study/result** | **D1** | **D2** | **D3** | **D4** | **D5** | **Overall** | **Result-specific basis** |
| --- | --- | --- | --- | --- | --- | --- | --- | --- |
| LVEF | Costanza 2015 | Some concerns | Some concerns | Some concerns | Low risk | Some concerns | Some concerns | 20 randomized; 16 analyzed; small pilot and incomplete attrition detail. |
| LVEF | Awoyemi 2021 | Some concerns | Some concerns | Some concerns | Some concerns | Some concerns | Some concerns | Open-label intervention; blinded endpoint; incomplete LVEF data. |
| LVEF | Ding 2021 | Some concerns | Some concerns | Low risk | Some concerns | Some concerns | Some concerns | Allocation concealment and blinding insufficiently described. |
| LVEF | Li 2024 | Some concerns | Some concerns | Some concerns | Some concerns | High risk | High risk | Unblinded report with incomplete prespecification and selective-reporting concern. |
| LVEF | Chen 2023 | Some concerns | Some concerns | High risk | Some concerns | Some concerns | High risk | Outcome available for imbalanced completers (44/36 of 50/50). |
| LVEF | Zhang 2017 | Some concerns | Some concerns | Low risk | Some concerns | Some concerns | Some concerns | Single-blind trial; allocation concealment incompletely reported. |
| LVEF | Zhou 2024 | Some concerns | High risk | High risk | Some concerns | Some concerns | High risk | Open-label thesis with substantial attrition and unclear missing-data handling. |
| NT-proBNP | Matin 2022/2024 | Some concerns | Low risk | Some concerns | Low risk | Some concerns | Some concerns | Linked reports; attrition and selective biomarker availability possible. |
| NT-proBNP | Pourrajab 2020/2022 | Some concerns | Low risk | Some concerns | Low risk | Some concerns | Some concerns | Triple-blind trial; some concerns from attrition/reporting; median/IQR conversion audited separately. |
| NT-proBNP | Chen 2023 | Some concerns | Some concerns | Some concerns | Some concerns | Some concerns | Some concerns | Imbalanced completer data and converted summary create some concerns. |
| NT-proBNP | Xu 2024 | Some concerns | Some concerns | Some concerns | Some concerns | High risk | High risk | Thesis result, converted summary, and no accessible prespecified analysis plan. |
| NT-proBNP | Zhou 2024 | Some concerns | High risk | High risk | Some concerns | Some concerns | High risk | Open-label thesis with substantial attrition and converted reporting. |
| CRP/hs-CRP | Costanza 2015 | Some concerns | Some concerns | Some concerns | Low risk | Some concerns | Some concerns | Small pilot with attrition; placebo-controlled biomarker measurement. |
| CRP/hs-CRP | Matin 2022/2024 | Some concerns | Low risk | Some concerns | Low risk | Some concerns | Some concerns | Linked reports; attrition and selective biomarker availability possible. |
| CRP/hs-CRP | Ding 2021 | Some concerns | Some concerns | Low risk | Some concerns | Some concerns | Some concerns | Allocation concealment and blinding insufficiently described. |
| CRP/hs-CRP | Xu 2024 | Some concerns | Some concerns | Some concerns | Some concerns | High risk | High risk | Thesis-derived converted result with selective-reporting concern. |
| CRP/hs-CRP | Yu 2021 (sensitivity) | Some concerns | Some concerns | Some concerns | High risk | High risk | High risk | Very small selected HF subgroup; result retained only for sensitivity/narrative use. |
| TNF-alpha | Ding 2021 | Some concerns | Some concerns | Low risk | Some concerns | Some concerns | Some concerns | Allocation concealment and blinding insufficiently described. |
| TNF-alpha | Li 2024 | Some concerns | Some concerns | Some concerns | Some concerns | Some concerns | Some concerns | Unblinded report; result-specific reporting judged some concerns. |
| TNF-alpha | Xu 2024 | Some concerns | Some concerns | Some concerns | Some concerns | High risk | High risk | Thesis-derived converted result with selective-reporting concern. |
| TNF-alpha | Yu 2021 (sensitivity) | Some concerns | Some concerns | Some concerns | High risk | High risk | High risk | Very small selected HF subgroup; result retained only for sensitivity/narrative use. |
| TMAO | Ding 2021 | Some concerns | Some concerns | Low risk | Some concerns | Some concerns | Some concerns | Allocation concealment and blinding insufficiently described. |
| TMAO | Li 2024 | Some concerns | Some concerns | Some concerns | Some concerns | Some concerns | Some concerns | Unblinded report; result-specific reporting judged some concerns. |
| TMAO | Chen 2023 | Some concerns | Some concerns | High risk | Some concerns | Some concerns | High risk | Imbalanced completer data (44/36) and converted summary. |
| 6MWT | Ding 2021 | Some concerns | Some concerns | Low risk | Some concerns | Some concerns | Some concerns | Allocation concealment and blinding insufficiently described. |
| 6MWT | Zhou 2024 | Some concerns | Some concerns | Some concerns | Some concerns | Some concerns | Some concerns | Open-label performance outcome and incomplete reporting; FAS result retained. |
| Readmission | Awoyemi 2021 | Some concerns | Some concerns | Low risk | Some concerns | Some concerns | Some concerns | Randomized arm comparison with blinded event review; open-label intervention. |
| Readmission | Li 2024 | Some concerns | Some concerns | Low risk | Some concerns | High risk | High risk | No blinding and incomplete prespecification of clinical-event reporting. |
| Readmission | Chen 2023 | Some concerns | Some concerns | Low risk | Some concerns | Some concerns | Some concerns | Randomized denominators retained; adjudication/blinding incompletely described. |
| Mortality | Matin 2022/2024 | Some concerns | Low risk | Low risk | Low risk | Some concerns | Some concerns | Deaths reported by randomized/completer groups; sparse event reporting. |
| Mortality | Li 2024 | Some concerns | Some concerns | Low risk | Some concerns | High risk | High risk | No blinding and incomplete prespecification of clinical-event reporting. |
| Mortality | Chen 2023 | Some concerns | Some concerns | Low risk | Some concerns | Some concerns | Some concerns | Randomized denominators retained; adjudication/blinding incompletely described. |
| Mortality | Zhou 2024 | Some concerns | High risk | Some concerns | Some concerns | Some concerns | High risk | Open-label trial with substantial attrition and unclear event follow-up completeness. |

These result-level judgments, rather than the descriptive study-level overview in Table S2.1, informed the CINeMA within-study-bias domain and the high-risk sensitivity analyses. The matrix covers every study-outcome result contributing to the principal primary or sensitivity syntheses. High-risk exclusions reproduce the sensitivity sets reported in Appendix 7; no statistical estimate changed in this audit.

# Appendix 3: Evidence geometry, heterogeneity, and inconsistency

The feasibility table distinguishes multi-node networks from two-node outcomes. Multi-node outcomes were analyzed as network meta-analyses. Outcomes labelled pairwise_meta_analysis_only had only one active node against control and are therefore ordinary random-effects pairwise meta-analyses generated through the same workflow, not true NMAs.

Table S3.1. Main-analysis network feasibility.

| **Outcome** | **Type** | **Measure** | **Analysis class** | **Studies** | **Nodes** | **Direct comparisons** | **Direct evidence** |
| --- | --- | --- | --- | --- | --- | --- | --- |
| LVEF | continuous | MD | network_meta_analysis | 7 | 3 | 2 | Control/placebo/usual care -- *S. boulardii*: 2; Control/placebo/usual care -- Multi-strain bacterial probiotic: 5 |
| NTproBNP | continuous | MD | network_meta_analysis | 5 | 4 | 3 | Control/placebo/usual care -- Multi-strain synbiotic: 1; Control/placebo/usual care -- Probiotic yogurt (La5/Bb12): 1; Control/placebo/usual care -- Multi-strain bacterial probiotic: 3 |
| CRP_hsCRP | continuous | SMD | network_meta_analysis | 4 | 4 | 3 | Control/placebo/usual care -- Multi-strain synbiotic: 1; Control/placebo/usual care -- *S. boulardii*: 1; Control/placebo/usual care -- Multi-strain bacterial probiotic: 2 |
| TNF_alpha | continuous | MD | pairwise_meta_analysis_only | 3 | 2 | 1 | Control/placebo/usual care -- Multi-strain bacterial probiotic: 3 |
| IL6 | continuous | MD | not_run | 1 | 2 | 1 | Control/placebo/usual care -- Multi-strain bacterial probiotic: 1 |
| TMAO | continuous | MD | pairwise_meta_analysis_only | 3 | 2 | 1 | Control/placebo/usual care -- Multi-strain bacterial probiotic: 3 |
| SixMWT | continuous | MD | pairwise_meta_analysis_only | 2 | 2 | 1 | Control/placebo/usual care -- Multi-strain bacterial probiotic: 2 |
| QoL_ADL | continuous | SMD | narrative_only | 3 | 2 | 1 | ADL, EQ-5D/VAS, and KCCQ were not pooled |
| IL8 | continuous | MD | not_run | 1 | 2 | 1 | Control/placebo/usual care -- Multi-strain bacterial probiotic: 1 |
| IL1beta | continuous | MD | not_run | 1 | 2 | 1 | Control/placebo/usual care -- Multi-strain bacterial probiotic: 1 |
| sCD163 | continuous | MD | network_meta_analysis | 2 | 3 | 2 | Control/placebo/usual care -- Multi-strain synbiotic: 1; Control/placebo/usual care -- Probiotic yogurt (La5/Bb12): 1 |
| sTWEAK | continuous | MD | network_meta_analysis | 2 | 3 | 2 | Control/placebo/usual care -- Multi-strain synbiotic: 1; Control/placebo/usual care -- Probiotic yogurt (La5/Bb12): 1 |
| LVESV | continuous | MD | not_run | 1 | 2 | 1 | Control/placebo/usual care -- Multi-strain bacterial probiotic: 1 |
| LVEDVI | continuous | MD | not_run | 1 | 2 | 1 | Control/placebo/usual care -- Multi-strain bacterial probiotic: 1 |
| LVESVI | continuous | MD | not_run | 1 | 2 | 1 | Control/placebo/usual care -- Multi-strain bacterial probiotic: 1 |
| ADMA | continuous | MD | not_run | 1 | 2 | 1 | Control/placebo/usual care -- Probiotic yogurt (La5/Bb12): 1 |
| LCAT | continuous | MD | not_run | 1 | 2 | 1 | Control/placebo/usual care -- Probiotic yogurt (La5/Bb12): 1 |
| BUN | continuous | MD | not_run | 1 | 2 | 1 | Control/placebo/usual care -- Probiotic yogurt (La5/Bb12): 1 |
| Readmission | binary | RR | network_meta_analysis | 3 | 3 | 2 | Control/placebo/usual care -- *S. boulardii*: 1; Control/placebo/usual care -- Multi-strain bacterial probiotic: 2 |
| Mortality | binary | RR | network_meta_analysis | 4 | 3 | 2 | Control/placebo/usual care -- Multi-strain synbiotic: 1; Control/placebo/usual care -- Multi-strain bacterial probiotic: 3 |
| Effective_rate | binary | RR | not_run | 1 | 2 | 1 | Control/placebo/usual care -- Multi-strain bacterial probiotic: 1 |
| Any_adverse_event | binary | RR | not_run | 1 | 2 | 1 | Control/placebo/usual care -- Multi-strain bacterial probiotic: 1 |

Table S3.2. Heterogeneity estimates for main analyses.

| **Outcome** | **tau** | **tau^2** | **I^2** | **Q** | **df** | **P value** |
| --- | --- | --- | --- | --- | --- | --- |
| LVEF | 2.15757668272025 | 4.65513714181812 | 0.736789056908083 | 18.9961706806921 | 5 | 0.00192529613918308 |
| NTproBNP | 260.839369639949 | 68037.176754166 | 0.788693190185303 | 9.46491029680432 | 2 | 0.0088048273235963 |
| CRP_hsCRP | 0.143424678114762 | 0.020570638292323 | 0.32137514578091 | 1.47356819276937 | 1 | 0.224783556041559 |
| TNF_alpha | 1.11095731531338 | 1.23422615644832 | 0.813848807089943 | 10.7439547860773 | 2 | 0.00464493735645533 |
| TMAO | 0.0239856446493374 | 0.000575311149244288 | 0.691385138891205 | 6.48056931806323 | 2 | 0.0391527483291236 |
| SixMWT | 0 | 0 | 0 | 0.183938616766318 | 1 | 0.668010078554788 |
| sCD163 | NA | NA | NA | 5.71152039082592e-34 | 0 | NA |
| sTWEAK | NA | NA | NA | 4.42801123588229e-26 | 0 | NA |
| Readmission | 0 | 0 | 0 | 0.00439603267466942 | 1 | 0.947136925841689 |
| Mortality | 0 | 0 | 0 | 1.73700555485706 | 2 | 0.419579282774664 |

Formal inconsistency statistics were not tabulated because the main evidence networks were sparse and control-centred, with no closed loops. Consequently, design-by-treatment, loop-specific, and node-splitting inconsistency tests were not meaningfully estimable; interpretation should therefore rely on evidence geometry, direct-comparison support, heterogeneity, and sensitivity analyses.

# Appendix 4: Multi-node network maps and NMA forest plots

The primary LVEF network map and forest plot are presented in manuscript Figure 2 and are not duplicated here. The figures below show only the remaining outcomes with three or more treatment nodes.

*Figure S4.1. N-terminal pro-B-type natriuretic peptide (NT-proBNP) network map, exploratory main broad-node analysis. Edges connect directly compared nodes; edge width and numeral denote the number of direct studies. The combined control node includes placebo, usual care, and the non-probiotic food comparator and is not assumed to be clinically exchangeable.*

*Figure S4.2. N-terminal pro-B-type natriuretic peptide (NT-proBNP) forest plot versus combined control, exploratory main broad-node analysis. Squares are mean differences (MDs), horizontal lines are 95% confidence intervals (CIs), and zero is the null value. The combined control node includes placebo, usual care, and the non-probiotic food comparator.*

*Figure S4.3. C-reactive protein/high-sensitivity C-reactive protein (CRP/hs-CRP) network map, exploratory main broad-node analysis. Edges connect directly compared nodes; edge width and numeral denote the number of direct studies. Saccharomyces boulardii is abbreviated as S. boulardii within the panel. The combined control node includes placebo and usual care.*

*Figure S4.4. C-reactive protein/high-sensitivity C-reactive protein (CRP/hs-CRP) forest plot versus combined control, exploratory main broad-node analysis. Squares are standardized mean differences (SMDs), horizontal lines are 95% confidence intervals (CIs), and zero is the null value. Saccharomyces boulardii is abbreviated as S. boulardii within the panel.*

*Figure S4.5. Soluble cluster of differentiation 163 (sCD163) network map, exploratory main broad-node analysis. Edges connect directly compared nodes; edge width and numeral denote the number of direct studies. The combined control node includes placebo, usual care, and the non-probiotic food comparator.*

*Figure S4.6. Soluble cluster of differentiation 163 (sCD163) forest plot versus combined control, exploratory main broad-node analysis. Squares are mean differences (MDs), horizontal lines are 95% confidence intervals (CIs), and zero is the null value.*

*Figure S4.7. Soluble tumor necrosis factor-like weak inducer of apoptosis (sTWEAK) network map, exploratory main broad-node analysis. Edges connect directly compared nodes; edge width and numeral denote the number of direct studies. The combined control node includes placebo, usual care, and the non-probiotic food comparator.*

*Figure S4.8. Soluble tumor necrosis factor-like weak inducer of apoptosis (sTWEAK) forest plot versus combined control, exploratory main broad-node analysis. Squares are mean differences (MDs), horizontal lines are 95% confidence intervals (CIs), and zero is the null value.*

*Figure S4.9. Readmission network map, exploratory main broad-node analysis. Edges connect directly compared nodes; edge width and numeral denote the number of direct studies. Saccharomyces boulardii is abbreviated as S. boulardii within the panel. The combined control node includes placebo and usual care.*

*Figure S4.10. Readmission forest plot versus combined control, exploratory main broad-node analysis. Squares are risk ratios (RRs), horizontal lines are 95% confidence intervals (CIs), and one is the null value. Saccharomyces boulardii is abbreviated as S. boulardii within the panel.*

*Figure S4.11. Mortality network map, exploratory main broad-node analysis. Edges connect directly compared nodes; edge width and numeral denote the number of direct studies. The combined control node includes placebo and usual care.*

*Figure S4.12. Mortality forest plot versus combined control, exploratory main broad-node analysis. Squares are risk ratios (RRs), horizontal lines are 95% confidence intervals (CIs), and one is the null value.*

# Appendix 5: Pairwise meta-analysis forest plots

The forest plots below show conventional DerSimonian-Laird pairwise analyses. Comparator-stratified REML Hartung-Knapp estimates are reported in Appendix 19 and take precedence for robustness interpretation. QoL/ADL was removed from quantitative pooling because ADL, EQ-5D/VAS, and KCCQ measure different constructs.

*Figure S5.1. Tumor necrosis factor-alpha (TNF-alpha) conventional pairwise random-effects analysis. The primary mean difference (MD) was -2.35 (95% confidence interval [CI] -3.78 to -0.92); squares show point estimates, horizontal lines show 95% CIs, and zero is the null value.*

*Figure S5.2. Trimethylamine N-oxide (TMAO) conventional pairwise random-effects analysis. Squares show mean differences (MDs), horizontal lines show 95% confidence intervals (CIs), and zero is the null value.*

*Figure S5.3. Six-minute walk test (6MWT) conventional pairwise random-effects analysis. Squares show mean differences (MDs) in meters, horizontal lines show 95% confidence intervals (CIs), and zero is the null value; Appendix 19 reports the Hartung-Knapp sensitivity.*

# Appendix 6: Descriptive P-scores for multi-node networks (not used for inference)

P-scores are retained only as descriptive outputs for transparency. Because the networks are sparse, star-shaped, and have no closed loops, P-scores cannot separate treatment effects from study, comparator, region, and formulation differences and are not evidence of superiority.

*Figure S6.1. Descriptive P-score heatmap for exploratory multi-node main broad-node analyses. Higher values and darker cells indicate higher statistical ranking; blank cells indicate that a treatment was absent from that outcome network. Outcomes are left ventricular ejection fraction (LVEF), N-terminal pro-B-type natriuretic peptide (NT-proBNP), C-reactive protein/high-sensitivity C-reactive protein (CRP/hs-CRP), soluble cluster of differentiation 163 (sCD163), and soluble tumor necrosis factor-like weak inducer of apoptosis (sTWEAK). Saccharomyces boulardii is abbreviated as S. boulardii. Rankings are not evidence of clinical superiority.*

Table S6.1. Descriptive P-scores for multi-node networks; these values are not used to rank treatments in the conclusions.

| **Treatment** | **LVEF** | **NT-proBNP** | **CRP/hs-CRP** | **sCD163** | **sTWEAK** | **Readmission** | **Mortality** |
| --- | --- | --- | --- | --- | --- | --- | --- |
| Multi-strain bacterial probiotic | 0.90 (rank 1) | 0.61 (rank 2) | 0.81 (rank 1) |  |  | 0.84 (rank 1) | 0.65 (rank 1) |
| Control | 0.30 (rank 2) | 0.33 (rank 4) | 0.20 (rank 3) | 0.26 (rank 3) | 0.61 (rank 1) | 0.16 (rank 3) | 0.39 (rank 3) |
| *S. boulardii* | 0.30 (rank 3) |  | 0.80 (rank 2) |  |  | 0.50 (rank 2) |  |
| Multi-strain synbiotic |  | 0.65 (rank 1) | 0.19 (rank 4) | 0.42 (rank 2) | 0.32 (rank 3) |  | 0.46 (rank 2) |
| Probiotic yogurt (La5/Bb12) |  | 0.41 (rank 3) |  | 0.81 (rank 1) | 0.57 (rank 2) |  |  |

# Appendix 7: League tables and estimates

League tables show estimable combined-comparator network outputs. They are exploratory because the comparator node is heterogeneous and no main network has a closed loop. QoL/ADL is not pooled.

## Table S7.1. LVEF league table

```
League table (random effects model):

 Control/placebo/usual care              -2.13 (-4.35; 0.10) 0.42 (-4.20; 5.04)
        -2.13 (-4.35; 0.10) Multi-strain bacterial probiotic                  .
         0.42 (-4.20; 5.04)               2.54 (-2.59; 7.67)       S. boulardii

Lower triangle: results from network meta-analysis (column vs row)
Upper triangle: results from direct comparisons (row vs column)
```

## Table S7.2. NT-proBNP league table

```
League table (random effects model):

 Control/placebo/usual care         122.99 (-209.77; 455.76)
   122.99 (-209.77; 455.76) Multi-strain bacterial probiotic
   175.54 (-417.17; 768.25)          52.55 (-627.19; 732.28)
    12.00 (-505.05; 529.05)        -110.99 (-725.86; 503.88)

  175.54 (-417.17; 768.25)     12.00 (-505.05; 529.05)
                         .                           .
    Multi-strain synbiotic                           .
 -163.54 (-950.08; 623.00) Probiotic yogurt (La5/Bb12)

Lower triangle: results from network meta-analysis (column vs row)
Upper triangle: results from direct comparisons (row vs column)
```

## Table S7.3. CRP/hs-CRP league table

```
League table (random effects model):

 Control/placebo/usual care              0.72 ( 0.37;  1.07)
        0.72 ( 0.37;  1.07) Multi-strain bacterial probiotic
       -0.01 (-0.53;  0.51)             -0.74 (-1.36; -0.11)
        0.80 (-0.26;  1.86)              0.08 (-1.04;  1.19)

   -0.01 (-0.53;  0.51) 0.80 (-0.26;  1.86)
                      .                   .
 Multi-strain synbiotic                   .
    0.81 (-0.37;  1.99)        S. boulardii

Lower triangle: results from network meta-analysis (column vs row)
Upper triangle: results from direct comparisons (row vs column)
```

## Table S7.4. TNF-alpha league table

```
League table (random effects model):

 Control/placebo/usual care              2.35 ( 0.92;  3.78)
        2.35 ( 0.92;  3.78) Multi-strain bacterial probiotic

Lower triangle: results from network meta-analysis (column vs row)
Upper triangle: results from direct comparisons (row vs column)
```

## Table S7.5. TMAO league table

```
League table (random effects model):

 Control/placebo/usual care              0.07 ( 0.03;  0.10)
        0.07 ( 0.03;  0.10) Multi-strain bacterial probiotic

Lower triangle: results from network meta-analysis (column vs row)
Upper triangle: results from direct comparisons (row vs column)
```

## Table S7.6. 6MWT league table

```
League table (random effects model):

 Control/placebo/usual care           -13.79 (-18.96; -8.63)
     -13.79 (-18.96; -8.63) Multi-strain bacterial probiotic

Lower triangle: results from network meta-analysis (column vs row)
Upper triangle: results from direct comparisons (row vs column)
```

## Table S7.8. sCD163 league table

```
League table (random effects model):

 Control/placebo/usual care     0.16 (-2.12; 2.44)          0.88 (-0.37; 2.14)
         0.16 (-2.12; 2.44) Multi-strain synbiotic                           .
         0.88 (-0.37; 2.14)     0.72 (-1.88; 3.32) Probiotic yogurt (La5/Bb12)

Lower triangle: results from network meta-analysis (column vs row)
Upper triangle: results from direct comparisons (row vs column)
```

## Table S7.9. sTWEAK league table

```
League table (random effects model):

 Control/placebo/usual care -98.26 (-476.80; 280.28)
   -98.26 (-476.80; 280.28)   Multi-strain synbiotic
    -6.67 (-169.67; 156.34)  91.59 (-320.56; 503.74)

     -6.67 (-169.67; 156.34)
                           .
 Probiotic yogurt (La5/Bb12)

Lower triangle: results from network meta-analysis (column vs row)
Upper triangle: results from direct comparisons (row vs column)
```

## Table S7.10. Readmission league table

```
League table (random effects model):

 Control/placebo/usual care                2.38 (1.32; 4.30) 1.53 (0.27; 8.77)
          2.38 (1.32; 4.30) Multi-strain bacterial probiotic                 .
          1.53 (0.27; 8.77)                0.64 (0.10; 4.05)      S. boulardii

Lower triangle: results from network meta-analysis (column vs row)
Upper triangle: results from direct comparisons (row vs column)
```

## Table S7.11. Mortality league table

```
League table (random effects model):

 Control/placebo/usual care               1.52 (0.34;  6.81)
         1.52 (0.34;  6.81) Multi-strain bacterial probiotic
         1.05 (0.07; 16.23)               0.69 (0.03; 15.60)

     1.05 (0.07; 16.23)
                      .
 Multi-strain synbiotic

Lower triangle: results from network meta-analysis (column vs row)
Upper triangle: results from direct comparisons (row vs column)
```

Table S7.12. Exploratory combined-comparator estimates; comparator-stratified robust sensitivities are reported in Table S19.1.

| **Outcome** | **Measure** | **Treatment** | **Comparator** | **Estimate (95% CI)** |
| --- | --- | --- | --- | --- |
| LVEF | MD | Multi-strain bacterial probiotic | Control/placebo/usual care | 2.13 (-0.10, 4.35) |
| LVEF | MD | *S. boulardii* | Control/placebo/usual care | -0.42 (-5.04, 4.20) |
| NTproBNP | MD | Multi-strain bacterial probiotic | Control/placebo/usual care | -122.99 (-455.76, 209.77) |
| NTproBNP | MD | Multi-strain synbiotic | Control/placebo/usual care | -175.54 (-768.25, 417.17) |
| NTproBNP | MD | Probiotic yogurt (La5/Bb12) | Control/placebo/usual care | -12.00 (-529.05, 505.05) |
| CRP_hsCRP | SMD | Multi-strain bacterial probiotic | Control/placebo/usual care | -0.72 (-1.07, -0.37) |
| CRP_hsCRP | SMD | Multi-strain synbiotic | Control/placebo/usual care | 0.01 (-0.51, 0.53) |
| CRP_hsCRP | SMD | *S. boulardii* | Control/placebo/usual care | -0.80 (-1.86, 0.26) |
| TNF_alpha | MD | Multi-strain bacterial probiotic | Control/placebo/usual care | -2.35 (-3.78, -0.92) |
| TMAO | MD | Multi-strain bacterial probiotic | Control/placebo/usual care | -0.07 (-0.10, -0.03) |
| SixMWT | MD | Multi-strain bacterial probiotic | Control/placebo/usual care | 13.79 (8.63, 18.96) |
| sCD163 | MD | Multi-strain synbiotic | Control/placebo/usual care | -0.16 (-2.44, 2.12) |
| sCD163 | MD | Probiotic yogurt (La5/Bb12) | Control/placebo/usual care | -0.88 (-2.14, 0.37) |
| sTWEAK | MD | Multi-strain synbiotic | Control/placebo/usual care | 98.26 (-280.28, 476.80) |
| sTWEAK | MD | Probiotic yogurt (La5/Bb12) | Control/placebo/usual care | 6.67 (-156.34, 169.67) |
| Readmission | RR | Multi-strain bacterial probiotic | Control/placebo/usual care | 0.42 (0.23, 0.76) |
| Readmission | RR | *S. boulardii* | Control/placebo/usual care | 0.65 (0.11, 3.75) |
| Mortality | RR | Multi-strain bacterial probiotic | Control/placebo/usual care | 0.66 (0.15, 2.93) |
| Mortality | RR | Multi-strain synbiotic | Control/placebo/usual care | 0.95 (0.06, 14.69) |

# Appendix 8: CINeMA certainty framework

Table S8.1. CINeMA domain-level considerations for final certainty assessment.

| **Domain** | **Current consideration** |
| --- | --- |
| Within-study bias | Informed by RoB 2 judgments in Appendix 2; several trials had some concerns or high risk. |
| Reporting bias | Formal small-study tests were not run because each network had fewer than 10 contrasts. |
| Indirectness | Nodes were clinically grouped; detailed formulation-level analyses are exploratory. |
| Imprecision | Major concern for most nodes because many comparisons were informed by one to three studies. |
| Heterogeneity | Moderate to high heterogeneity was observed for several continuous outcomes. |
| Incoherence | No closed loops were present in main networks; incoherence was generally non-estimable. |

# Appendix 9: Feasibility of assessing small-study effects

Every outcome contained fewer than 10 contrasts. Funnel plots and Egger/Begg tests were therefore not produced because visual asymmetry and formal tests would be unreliable. Table S9.1 records this feasibility decision.

Table S9.1. Small-study-effect assessment feasibility; no formal test or funnel plot was interpreted.

| **Outcome** | **Analysis** | **Contrasts** | **Egger P** | **Begg P** | **Note** |
| --- | --- | --- | --- | --- | --- |
| LVEF | Primary_broad_control | 7 | NA | NA | Not tested because fewer than 10 contrasts. |
| NTproBNP | Primary_broad_control | 5 | NA | NA | Not tested because fewer than 10 contrasts. |
| CRP_hsCRP | Primary_broad_control | 4 | NA | NA | Not tested because fewer than 10 contrasts. |
| TNF_alpha | Primary_broad_control | 3 | NA | NA | Not tested because fewer than 10 contrasts. |
| TMAO | Primary_broad_control | 3 | NA | NA | Not tested because fewer than 10 contrasts. |
| SixMWT | Primary_broad_control | 2 | NA | NA | Not tested because fewer than 10 contrasts. |
| QoL_ADL | Primary_broad_control | 3 | NA | NA | Not tested because fewer than 10 contrasts. |
| sCD163 | Primary_broad_control | 2 | NA | NA | Not tested because fewer than 10 contrasts. |
| sTWEAK | Primary_broad_control | 2 | NA | NA | Not tested because fewer than 10 contrasts. |
| Readmission | Primary_broad_control | 3 | NA | NA | Not tested because fewer than 10 contrasts. |
| Mortality | Primary_broad_control | 4 | NA | NA | Not tested because fewer than 10 contrasts. |

# Appendix 10: Sensitivity analyses

Table S10.1. Sensitivity-analysis feasibility.

| **Outcome** | **Sensitivity analysis** | **Analysis class** | **Studies** | **Nodes** | **Direct comparisons** |
| --- | --- | --- | --- | --- | --- |
| LVEF | Sensitivity_excluding_theses | network_meta_analysis | 6 | 3 | 2 |
| LVEF | Sensitivity_excluding_open_label | network_meta_analysis | 5 | 3 | 2 |
| LVEF | Sensitivity_excluding_small_subgroup | network_meta_analysis | 7 | 3 | 2 |
| NTproBNP | Sensitivity_excluding_theses | network_meta_analysis | 3 | 4 | 3 |
| NTproBNP | Sensitivity_excluding_open_label | network_meta_analysis | 4 | 4 | 3 |
| NTproBNP | Sensitivity_excluding_small_subgroup | network_meta_analysis | 5 | 4 | 3 |
| CRP_hsCRP | Sensitivity_excluding_theses | network_meta_analysis | 3 | 4 | 3 |
| CRP_hsCRP | Sensitivity_excluding_open_label | network_meta_analysis | 4 | 4 | 3 |
| CRP_hsCRP | Sensitivity_excluding_small_subgroup | network_meta_analysis | 4 | 4 | 3 |
| Readmission | Sensitivity_excluding_theses | network_meta_analysis | 3 | 3 | 2 |
| Readmission | Sensitivity_excluding_open_label | pairwise_meta_analysis_only | 2 | 2 | 1 |
| Readmission | Sensitivity_excluding_small_subgroup | network_meta_analysis | 3 | 3 | 2 |
| Mortality | Sensitivity_excluding_theses | network_meta_analysis | 3 | 3 | 2 |
| Mortality | Sensitivity_excluding_open_label | network_meta_analysis | 3 | 3 | 2 |
| Mortality | Sensitivity_excluding_small_subgroup | network_meta_analysis | 4 | 3 | 2 |
| LVEF | Sensitivity_strict_control_excluding_ordinary_yogurt | network_meta_analysis | 7 | 3 | 2 |
| NTproBNP | Sensitivity_strict_control_excluding_ordinary_yogurt | network_meta_analysis | 4 | 3 | 2 |
| CRP_hsCRP | Sensitivity_strict_control_excluding_ordinary_yogurt | network_meta_analysis | 4 | 4 | 3 |
| TNF_alpha | Sensitivity_strict_control_excluding_ordinary_yogurt | pairwise_meta_analysis_only | 3 | 2 | 1 |
| IL6 | Sensitivity_strict_control_excluding_ordinary_yogurt | not_run | 1 | 2 | 1 |
| TMAO | Sensitivity_strict_control_excluding_ordinary_yogurt | pairwise_meta_analysis_only | 3 | 2 | 1 |
| SixMWT | Sensitivity_strict_control_excluding_ordinary_yogurt | pairwise_meta_analysis_only | 2 | 2 | 1 |
| QoL_ADL | Sensitivity_strict_control_excluding_ordinary_yogurt | pairwise_meta_analysis_only | 3 | 2 | 1 |
| IL8 | Sensitivity_strict_control_excluding_ordinary_yogurt | not_run | 1 | 2 | 1 |
| IL1beta | Sensitivity_strict_control_excluding_ordinary_yogurt | not_run | 1 | 2 | 1 |
| sCD163 | Sensitivity_strict_control_excluding_ordinary_yogurt | not_run | 1 | 2 | 1 |
| sTWEAK | Sensitivity_strict_control_excluding_ordinary_yogurt | not_run | 1 | 2 | 1 |
| LVESV | Sensitivity_strict_control_excluding_ordinary_yogurt | not_run | 1 | 2 | 1 |
| LVEDVI | Sensitivity_strict_control_excluding_ordinary_yogurt | not_run | 1 | 2 | 1 |
| LVESVI | Sensitivity_strict_control_excluding_ordinary_yogurt | not_run | 1 | 2 | 1 |
| ADMA | Sensitivity_strict_control_excluding_ordinary_yogurt | not_run | 0 | 0 | 0 |
| LCAT | Sensitivity_strict_control_excluding_ordinary_yogurt | not_run | 0 | 0 | 0 |
| BUN | Sensitivity_strict_control_excluding_ordinary_yogurt | not_run | 0 | 0 | 0 |
| Readmission | Sensitivity_strict_control_excluding_ordinary_yogurt | network_meta_analysis | 3 | 3 | 2 |
| Mortality | Sensitivity_strict_control_excluding_ordinary_yogurt | network_meta_analysis | 4 | 3 | 2 |
| Effective_rate | Sensitivity_strict_control_excluding_ordinary_yogurt | not_run | 1 | 2 | 1 |
| Any_adverse_event | Sensitivity_strict_control_excluding_ordinary_yogurt | not_run | 1 | 2 | 1 |
| LVEF | Sensitivity_including_Yu2021 | network_meta_analysis | 7 | 3 | 2 |
| LVEF | Sensitivity_excluding_converted_values | network_meta_analysis | 7 | 3 | 2 |
| LVEF | Sensitivity_excluding_high_risk_RoB2 | network_meta_analysis | 4 | 3 | 2 |
| NTproBNP | Sensitivity_including_Yu2021 | network_meta_analysis | 5 | 4 | 3 |
| NTproBNP | Sensitivity_excluding_converted_values | not_run | 1 | 2 | 1 |
| NTproBNP | Sensitivity_excluding_high_risk_RoB2 | network_meta_analysis | 3 | 4 | 3 |
| CRP_hsCRP | Sensitivity_including_Yu2021 | network_meta_analysis | 5 | 4 | 3 |
| CRP_hsCRP | Sensitivity_excluding_converted_values | network_meta_analysis | 3 | 4 | 3 |
| CRP_hsCRP | Sensitivity_excluding_high_risk_RoB2 | network_meta_analysis | 3 | 4 | 3 |
| TNF_alpha | Sensitivity_including_Yu2021 | pairwise_meta_analysis_only | 4 | 2 | 1 |
| TNF_alpha | Sensitivity_excluding_converted_values | pairwise_meta_analysis_only | 2 | 2 | 1 |
| TNF_alpha | Sensitivity_excluding_high_risk_RoB2 | pairwise_meta_analysis_only | 2 | 2 | 1 |
| IL6 | Sensitivity_including_Yu2021 | pairwise_meta_analysis_only | 2 | 2 | 1 |
| IL6 | Sensitivity_excluding_converted_values | not_run | 1 | 2 | 1 |
| IL6 | Sensitivity_excluding_high_risk_RoB2 | not_run | 1 | 2 | 1 |
| TMAO | Sensitivity_including_Yu2021 | pairwise_meta_analysis_only | 3 | 2 | 1 |
| TMAO | Sensitivity_excluding_converted_values | pairwise_meta_analysis_only | 2 | 2 | 1 |
| TMAO | Sensitivity_excluding_high_risk_RoB2 | pairwise_meta_analysis_only | 2 | 2 | 1 |
| SixMWT | Sensitivity_including_Yu2021 | pairwise_meta_analysis_only | 2 | 2 | 1 |
| SixMWT | Sensitivity_excluding_converted_values | pairwise_meta_analysis_only | 2 | 2 | 1 |
| SixMWT | Sensitivity_excluding_high_risk_RoB2 | pairwise_meta_analysis_only | 2 | 2 | 1 |
| QoL_ADL | Sensitivity_including_Yu2021 | pairwise_meta_analysis_only | 3 | 2 | 1 |
| QoL_ADL | Sensitivity_excluding_converted_values | pairwise_meta_analysis_only | 3 | 2 | 1 |
| QoL_ADL | Sensitivity_excluding_high_risk_RoB2 | pairwise_meta_analysis_only | 3 | 2 | 1 |
| IL8 | Sensitivity_including_Yu2021 | pairwise_meta_analysis_only | 2 | 2 | 1 |
| IL8 | Sensitivity_excluding_converted_values | not_run | 1 | 2 | 1 |
| IL8 | Sensitivity_excluding_high_risk_RoB2 | not_run | 1 | 2 | 1 |
| IL1beta | Sensitivity_including_Yu2021 | not_run | 1 | 2 | 1 |
| IL1beta | Sensitivity_excluding_converted_values | not_run | 1 | 2 | 1 |
| IL1beta | Sensitivity_excluding_high_risk_RoB2 | not_run | 1 | 2 | 1 |
| sCD163 | Sensitivity_including_Yu2021 | network_meta_analysis | 2 | 3 | 2 |
| sCD163 | Sensitivity_excluding_converted_values | not_run | 1 | 2 | 1 |
| sCD163 | Sensitivity_excluding_high_risk_RoB2 | network_meta_analysis | 2 | 3 | 2 |
| sTWEAK | Sensitivity_including_Yu2021 | network_meta_analysis | 2 | 3 | 2 |
| sTWEAK | Sensitivity_excluding_converted_values | not_run | 1 | 2 | 1 |
| sTWEAK | Sensitivity_excluding_high_risk_RoB2 | network_meta_analysis | 2 | 3 | 2 |
| LVESV | Sensitivity_including_Yu2021 | not_run | 1 | 2 | 1 |
| LVESV | Sensitivity_excluding_converted_values | not_run | 1 | 2 | 1 |
| LVESV | Sensitivity_excluding_high_risk_RoB2 | not_run | 1 | 2 | 1 |
| LVEDVI | Sensitivity_including_Yu2021 | not_run | 1 | 2 | 1 |
| LVEDVI | Sensitivity_excluding_converted_values | not_run | 1 | 2 | 1 |
| LVEDVI | Sensitivity_excluding_high_risk_RoB2 | not_run | 1 | 2 | 1 |
| LVESVI | Sensitivity_including_Yu2021 | not_run | 1 | 2 | 1 |
| LVESVI | Sensitivity_excluding_converted_values | not_run | 1 | 2 | 1 |
| LVESVI | Sensitivity_excluding_high_risk_RoB2 | not_run | 1 | 2 | 1 |
| ADMA | Sensitivity_including_Yu2021 | not_run | 1 | 2 | 1 |
| ADMA | Sensitivity_excluding_converted_values | not_run | 0 | 0 | 0 |
| ADMA | Sensitivity_excluding_high_risk_RoB2 | not_run | 1 | 2 | 1 |
| LCAT | Sensitivity_including_Yu2021 | not_run | 1 | 2 | 1 |
| LCAT | Sensitivity_excluding_converted_values | not_run | 0 | 0 | 0 |
| LCAT | Sensitivity_excluding_high_risk_RoB2 | not_run | 1 | 2 | 1 |
| BUN | Sensitivity_including_Yu2021 | not_run | 1 | 2 | 1 |
| BUN | Sensitivity_excluding_converted_values | not_run | 0 | 0 | 0 |
| BUN | Sensitivity_excluding_high_risk_RoB2 | not_run | 1 | 2 | 1 |
| Readmission | Sensitivity_including_Yu2021 | network_meta_analysis | 3 | 3 | 2 |
| Readmission | Sensitivity_excluding_converted_values | network_meta_analysis | 3 | 3 | 2 |
| Readmission | Sensitivity_excluding_high_risk_RoB2 | network_meta_analysis | 2 | 3 | 2 |
| Mortality | Sensitivity_including_Yu2021 | network_meta_analysis | 4 | 3 | 2 |
| Mortality | Sensitivity_excluding_converted_values | network_meta_analysis | 4 | 3 | 2 |
| Mortality | Sensitivity_excluding_high_risk_RoB2 | network_meta_analysis | 2 | 3 | 2 |
| Effective_rate | Sensitivity_including_Yu2021 | not_run | 1 | 2 | 1 |
| Effective_rate | Sensitivity_excluding_converted_values | not_run | 1 | 2 | 1 |
| Effective_rate | Sensitivity_excluding_high_risk_RoB2 | not_run | 1 | 2 | 1 |
| Any_adverse_event | Sensitivity_including_Yu2021 | not_run | 1 | 2 | 1 |
| Any_adverse_event | Sensitivity_excluding_converted_values | not_run | 1 | 2 | 1 |
| Any_adverse_event | Sensitivity_excluding_high_risk_RoB2 | not_run | 1 | 2 | 1 |

Table S10.2. Sensitivity-analysis estimates versus control.

| **Outcome** | **Sensitivity analysis** | **Measure** | **Treatment** | **Estimate (95% CI)** |
| --- | --- | --- | --- | --- |
| LVEF | Sensitivity_excluding_theses | MD | Multi-strain bacterial probiotic | 2.68 (-0.33, 5.68) |
| LVEF | Sensitivity_excluding_theses | MD | *S. boulardii* | -0.29 (-5.52, 4.94) |
| LVEF | Sensitivity_excluding_open_label | MD | Multi-strain bacterial probiotic | 2.69 (-0.43, 5.81) |
| LVEF | Sensitivity_excluding_open_label | MD | *S. boulardii* | 2.30 (-8.08, 12.68) |
| LVEF | Sensitivity_excluding_small_subgroup | MD | Multi-strain bacterial probiotic | 2.13 (-0.10, 4.35) |
| LVEF | Sensitivity_excluding_small_subgroup | MD | *S. boulardii* | -0.42 (-5.04, 4.20) |
| NTproBNP | Sensitivity_excluding_theses | MD | Multi-strain bacterial probiotic | 190.00 (-46.75, 426.75) |
| NTproBNP | Sensitivity_excluding_theses | MD | Multi-strain synbiotic | -175.54 (-475.45, 124.37) |
| NTproBNP | Sensitivity_excluding_theses | MD | Probiotic yogurt (La5/Bb12) | -12.00 (-89.30, 65.30) |
| NTproBNP | Sensitivity_excluding_open_label | MD | Multi-strain bacterial probiotic | -17.28 (-430.72, 396.16) |
| NTproBNP | Sensitivity_excluding_open_label | MD | Multi-strain synbiotic | -175.54 (-783.71, 432.63) |
| NTproBNP | Sensitivity_excluding_open_label | MD | Probiotic yogurt (La5/Bb12) | -12.00 (-546.70, 522.70) |
| NTproBNP | Sensitivity_excluding_small_subgroup | MD | Multi-strain bacterial probiotic | -122.99 (-455.76, 209.77) |
| NTproBNP | Sensitivity_excluding_small_subgroup | MD | Multi-strain synbiotic | -175.54 (-768.25, 417.17) |
| NTproBNP | Sensitivity_excluding_small_subgroup | MD | Probiotic yogurt (La5/Bb12) | -12.00 (-529.05, 505.05) |
| CRP_hsCRP | Sensitivity_excluding_theses | SMD | Multi-strain bacterial probiotic | -0.90 (-1.32, -0.49) |
| CRP_hsCRP | Sensitivity_excluding_theses | SMD | Multi-strain synbiotic | 0.01 (-0.43, 0.45) |
| CRP_hsCRP | Sensitivity_excluding_theses | SMD | *S. boulardii* | -0.80 (-1.82, 0.22) |
| CRP_hsCRP | Sensitivity_excluding_open_label | SMD | Multi-strain bacterial probiotic | -0.72 (-1.07, -0.37) |
| CRP_hsCRP | Sensitivity_excluding_open_label | SMD | Multi-strain synbiotic | 0.01 (-0.51, 0.53) |
| CRP_hsCRP | Sensitivity_excluding_open_label | SMD | *S. boulardii* | -0.80 (-1.86, 0.26) |
| CRP_hsCRP | Sensitivity_excluding_small_subgroup | SMD | Multi-strain bacterial probiotic | -0.72 (-1.07, -0.37) |
| CRP_hsCRP | Sensitivity_excluding_small_subgroup | SMD | Multi-strain synbiotic | 0.01 (-0.51, 0.53) |
| CRP_hsCRP | Sensitivity_excluding_small_subgroup | SMD | *S. boulardii* | -0.80 (-1.86, 0.26) |
| Readmission | Sensitivity_excluding_theses | RR | Multi-strain bacterial probiotic | 0.42 (0.23, 0.76) |
| Readmission | Sensitivity_excluding_theses | RR | *S. boulardii* | 0.65 (0.11, 3.75) |
| Readmission | Sensitivity_excluding_open_label | RR | Multi-strain bacterial probiotic | 0.42 (0.23, 0.76) |
| Readmission | Sensitivity_excluding_small_subgroup | RR | Multi-strain bacterial probiotic | 0.42 (0.23, 0.76) |
| Readmission | Sensitivity_excluding_small_subgroup | RR | *S. boulardii* | 0.65 (0.11, 3.75) |
| Mortality | Sensitivity_excluding_theses | RR | Multi-strain bacterial probiotic | 0.65 (0.06, 6.98) |
| Mortality | Sensitivity_excluding_theses | RR | Multi-strain synbiotic | 0.95 (0.03, 30.27) |
| Mortality | Sensitivity_excluding_open_label | RR | Multi-strain bacterial probiotic | 0.65 (0.06, 6.98) |
| Mortality | Sensitivity_excluding_open_label | RR | Multi-strain synbiotic | 0.95 (0.03, 30.27) |
| Mortality | Sensitivity_excluding_small_subgroup | RR | Multi-strain bacterial probiotic | 0.66 (0.15, 2.93) |
| Mortality | Sensitivity_excluding_small_subgroup | RR | Multi-strain synbiotic | 0.95 (0.06, 14.69) |
| LVEF | Sensitivity_strict_control_excluding_ordinary_yogurt | MD | Multi-strain bacterial probiotic | 2.13 (-0.10, 4.35) |
| LVEF | Sensitivity_strict_control_excluding_ordinary_yogurt | MD | *S. boulardii* | -0.42 (-5.04, 4.20) |
| NTproBNP | Sensitivity_strict_control_excluding_ordinary_yogurt | MD | Multi-strain bacterial probiotic | -122.99 (-455.76, 209.77) |
| NTproBNP | Sensitivity_strict_control_excluding_ordinary_yogurt | MD | Multi-strain synbiotic | -175.54 (-768.25, 417.17) |
| CRP_hsCRP | Sensitivity_strict_control_excluding_ordinary_yogurt | SMD | Multi-strain bacterial probiotic | -0.72 (-1.07, -0.37) |
| CRP_hsCRP | Sensitivity_strict_control_excluding_ordinary_yogurt | SMD | Multi-strain synbiotic | 0.01 (-0.51, 0.53) |
| CRP_hsCRP | Sensitivity_strict_control_excluding_ordinary_yogurt | SMD | *S. boulardii* | -0.80 (-1.86, 0.26) |
| TNF_alpha | Sensitivity_strict_control_excluding_ordinary_yogurt | MD | Multi-strain bacterial probiotic | -2.35 (-3.78, -0.92) |
| TMAO | Sensitivity_strict_control_excluding_ordinary_yogurt | MD | Multi-strain bacterial probiotic | -0.07 (-0.10, -0.03) |
| SixMWT | Sensitivity_strict_control_excluding_ordinary_yogurt | MD | Multi-strain bacterial probiotic | 13.79 (8.63, 18.96) |
| QoL_ADL | Sensitivity_strict_control_excluding_ordinary_yogurt | SMD | Multi-strain bacterial probiotic | 0.73 (0.15, 1.30) |
| Readmission | Sensitivity_strict_control_excluding_ordinary_yogurt | RR | Multi-strain bacterial probiotic | 0.42 (0.23, 0.76) |
| Readmission | Sensitivity_strict_control_excluding_ordinary_yogurt | RR | *S. boulardii* | 0.65 (0.11, 3.75) |
| Mortality | Sensitivity_strict_control_excluding_ordinary_yogurt | RR | Multi-strain bacterial probiotic | 0.66 (0.15, 2.93) |
| Mortality | Sensitivity_strict_control_excluding_ordinary_yogurt | RR | Multi-strain synbiotic | 0.95 (0.06, 14.69) |
| LVEF | Sensitivity_including_Yu2021 | MD | Multi-strain bacterial probiotic | 2.13 (-0.10, 4.35) |
| LVEF | Sensitivity_including_Yu2021 | MD | *S. boulardii* | -0.42 (-5.04, 4.20) |
| LVEF | Sensitivity_excluding_converted_values | MD | Multi-strain bacterial probiotic | 2.13 (-0.10, 4.35) |
| LVEF | Sensitivity_excluding_converted_values | MD | *S. boulardii* | -0.42 (-5.04, 4.20) |
| LVEF | Sensitivity_excluding_high_risk_RoB2 | MD | Multi-strain bacterial probiotic | 1.06 (-3.35, 5.48) |
| LVEF | Sensitivity_excluding_high_risk_RoB2 | MD | *S. boulardii* | -0.29 (-5.53, 4.96) |
| NTproBNP | Sensitivity_including_Yu2021 | MD | Multi-strain bacterial probiotic | -122.99 (-455.76, 209.77) |
| NTproBNP | Sensitivity_including_Yu2021 | MD | Multi-strain synbiotic | -175.54 (-768.25, 417.17) |
| NTproBNP | Sensitivity_including_Yu2021 | MD | Probiotic yogurt (La5/Bb12) | -12.00 (-529.05, 505.05) |
| NTproBNP | Sensitivity_excluding_high_risk_RoB2 | MD | Multi-strain bacterial probiotic | 190.00 (-46.75, 426.75) |
| NTproBNP | Sensitivity_excluding_high_risk_RoB2 | MD | Multi-strain synbiotic | -175.54 (-475.45, 124.37) |
| NTproBNP | Sensitivity_excluding_high_risk_RoB2 | MD | Probiotic yogurt (La5/Bb12) | -12.00 (-89.30, 65.30) |
| CRP_hsCRP | Sensitivity_including_Yu2021 | SMD | Multi-strain bacterial probiotic | -0.83 (-1.30, -0.36) |
| CRP_hsCRP | Sensitivity_including_Yu2021 | SMD | Multi-strain synbiotic | 0.01 (-0.70, 0.72) |
| CRP_hsCRP | Sensitivity_including_Yu2021 | SMD | *S. boulardii* | -0.80 (-1.96, 0.36) |
| CRP_hsCRP | Sensitivity_excluding_converted_values | SMD | Multi-strain bacterial probiotic | -0.90 (-1.32, -0.49) |
| CRP_hsCRP | Sensitivity_excluding_converted_values | SMD | Multi-strain synbiotic | 0.01 (-0.43, 0.45) |
| CRP_hsCRP | Sensitivity_excluding_converted_values | SMD | *S. boulardii* | -0.80 (-1.82, 0.22) |
| CRP_hsCRP | Sensitivity_excluding_high_risk_RoB2 | SMD | Multi-strain bacterial probiotic | -0.90 (-1.32, -0.49) |
| CRP_hsCRP | Sensitivity_excluding_high_risk_RoB2 | SMD | Multi-strain synbiotic | 0.01 (-0.43, 0.45) |
| CRP_hsCRP | Sensitivity_excluding_high_risk_RoB2 | SMD | *S. boulardii* | -0.80 (-1.82, 0.22) |
| TNF_alpha | Sensitivity_including_Yu2021 | MD | Multi-strain bacterial probiotic | -3.28 (-4.98, -1.58) |
| TNF_alpha | Sensitivity_excluding_converted_values | MD | Multi-strain bacterial probiotic | -3.17 (-3.57, -2.76) |
| TNF_alpha | Sensitivity_excluding_high_risk_RoB2 | MD | Multi-strain bacterial probiotic | -3.17 (-3.57, -2.76) |
| IL6 | Sensitivity_including_Yu2021 | MD | Multi-strain bacterial probiotic | -0.74 (-1.78, 0.30) |
| TMAO | Sensitivity_including_Yu2021 | MD | Multi-strain bacterial probiotic | -0.07 (-0.10, -0.03) |
| TMAO | Sensitivity_excluding_converted_values | MD | Multi-strain bacterial probiotic | -0.07 (-0.10, -0.04) |
| TMAO | Sensitivity_excluding_high_risk_RoB2 | MD | Multi-strain bacterial probiotic | -0.07 (-0.10, -0.04) |
| SixMWT | Sensitivity_including_Yu2021 | MD | Multi-strain bacterial probiotic | 13.79 (8.63, 18.96) |
| SixMWT | Sensitivity_excluding_converted_values | MD | Multi-strain bacterial probiotic | 13.79 (8.63, 18.96) |
| SixMWT | Sensitivity_excluding_high_risk_RoB2 | MD | Multi-strain bacterial probiotic | 13.79 (8.63, 18.96) |
| QoL_ADL | Sensitivity_including_Yu2021 | SMD | Multi-strain bacterial probiotic | 0.73 (0.15, 1.30) |
| QoL_ADL | Sensitivity_excluding_converted_values | SMD | Multi-strain bacterial probiotic | 0.73 (0.15, 1.30) |
| QoL_ADL | Sensitivity_excluding_high_risk_RoB2 | SMD | Multi-strain bacterial probiotic | 0.73 (0.15, 1.30) |
| IL8 | Sensitivity_including_Yu2021 | MD | Multi-strain bacterial probiotic | -2.44 (-3.98, -0.90) |
| sCD163 | Sensitivity_including_Yu2021 | MD | Multi-strain synbiotic | -0.16 (-2.44, 2.12) |
| sCD163 | Sensitivity_including_Yu2021 | MD | Probiotic yogurt (La5/Bb12) | -0.88 (-2.14, 0.37) |
| sCD163 | Sensitivity_excluding_high_risk_RoB2 | MD | Multi-strain synbiotic | -0.16 (-2.44, 2.12) |
| sCD163 | Sensitivity_excluding_high_risk_RoB2 | MD | Probiotic yogurt (La5/Bb12) | -0.88 (-2.14, 0.37) |
| sTWEAK | Sensitivity_including_Yu2021 | MD | Multi-strain synbiotic | 98.26 (-280.28, 476.80) |
| sTWEAK | Sensitivity_including_Yu2021 | MD | Probiotic yogurt (La5/Bb12) | 6.67 (-156.34, 169.67) |
| sTWEAK | Sensitivity_excluding_high_risk_RoB2 | MD | Multi-strain synbiotic | 98.26 (-280.28, 476.80) |
| sTWEAK | Sensitivity_excluding_high_risk_RoB2 | MD | Probiotic yogurt (La5/Bb12) | 6.67 (-156.34, 169.67) |
| Readmission | Sensitivity_including_Yu2021 | RR | Multi-strain bacterial probiotic | 0.42 (0.23, 0.76) |
| Readmission | Sensitivity_including_Yu2021 | RR | *S. boulardii* | 0.65 (0.11, 3.75) |
| Readmission | Sensitivity_excluding_converted_values | RR | Multi-strain bacterial probiotic | 0.42 (0.23, 0.76) |
| Readmission | Sensitivity_excluding_converted_values | RR | *S. boulardii* | 0.65 (0.11, 3.75) |
| Readmission | Sensitivity_excluding_high_risk_RoB2 | RR | Multi-strain bacterial probiotic | 0.43 (0.18, 1.03) |
| Readmission | Sensitivity_excluding_high_risk_RoB2 | RR | *S. boulardii* | 0.65 (0.11, 3.75) |
| Mortality | Sensitivity_including_Yu2021 | RR | Multi-strain bacterial probiotic | 0.66 (0.15, 2.93) |
| Mortality | Sensitivity_including_Yu2021 | RR | Multi-strain synbiotic | 0.95 (0.06, 14.69) |
| Mortality | Sensitivity_excluding_converted_values | RR | Multi-strain bacterial probiotic | 0.66 (0.15, 2.93) |
| Mortality | Sensitivity_excluding_converted_values | RR | Multi-strain synbiotic | 0.95 (0.06, 14.69) |
| Mortality | Sensitivity_excluding_high_risk_RoB2 | RR | Multi-strain bacterial probiotic | 3.00 (0.13, 71.91) |
| Mortality | Sensitivity_excluding_high_risk_RoB2 | RR | Multi-strain synbiotic | 0.95 (0.06, 14.69) |

# Appendix 11: Meta-regression feasibility

Meta-regression was not performed. The largest synthesis contained only seven contrasts, and treatment, comparator, region, and duration were strongly confounded; numerical coefficients from such models would be unstable and potentially misleading.

# Appendix 12: Exploratory detailed formulation-level analyses

Table S12.1. Detailed formulation-level feasibility.

| **Outcome** | **Analysis class** | **Studies** | **Nodes** | **Direct comparisons** | **Direct evidence** |
| --- | --- | --- | --- | --- | --- |
| LVEF | network_meta_analysis | 7 | 6 | 5 | Bifidobacterium triple viable -- Control/placebo/usual care: 1; Control/placebo/usual care -- Jinshuangqi: 1; Control/placebo/usual care -- Triple live bacteria: 1; Bifidobacterium viable capsules -- Control/placebo/usual care: 2; Control/placebo/usual care -- *S. boulardii*: 2 |
| NTproBNP | network_meta_analysis | 5 | 6 | 5 | Bifidobacterium quadruple viable -- Control/placebo/usual care: 1; Bifidobacterium triple viable -- Control/placebo/usual care: 1; Control/placebo/usual care -- Multi-strain synbiotic: 1; Control/placebo/usual care -- Probiotic yogurt (La5/Bb12): 1; Control/placebo/usual care -- Triple live bacteria: 1 |
| CRP_hsCRP | network_meta_analysis | 4 | 5 | 4 | Bifidobacterium quadruple viable -- Control/placebo/usual care: 1; Control/placebo/usual care -- Jinshuangqi: 1; Control/placebo/usual care -- Multi-strain synbiotic: 1; Control/placebo/usual care -- *S. boulardii*: 1 |
| Readmission | network_meta_analysis | 3 | 4 | 3 | Bifidobacterium triple viable -- Control/placebo/usual care: 1; Bifidobacterium viable capsules -- Control/placebo/usual care: 1; Control/placebo/usual care -- *S. boulardii*: 1 |
| Mortality | network_meta_analysis | 4 | 5 | 4 | Bifidobacterium triple viable -- Control/placebo/usual care: 1; Bifidobacterium viable capsules -- Control/placebo/usual care: 1; Control/placebo/usual care -- Multi-strain synbiotic: 1; Control/placebo/usual care -- Triple live bacteria: 1 |

Table S12.2. Detailed formulation-level estimates versus control.

| **Outcome** | **Measure** | **Detailed node** | **Estimate versus control (95% CI)** |
| --- | --- | --- | --- |
| LVEF | MD | Bifidobacterium triple viable | 3.42 (0.43, 6.41) |
| LVEF | MD | Bifidobacterium viable capsules | 4.61 (2.69, 6.54) |
| LVEF | MD | Jinshuangqi | -1.06 (-3.23, 1.11) |
| LVEF | MD | *S. boulardii* | -0.81 (-3.74, 2.11) |
| LVEF | MD | Triple live bacteria | 0.60 (-0.83, 2.03) |
| NTproBNP | MD | Bifidobacterium quadruple viable | -231.95 (-492.76, 28.87) |
| NTproBNP | MD | Bifidobacterium triple viable | 190.00 (-46.75, 426.75) |
| NTproBNP | MD | Multi-strain synbiotic | -175.54 (-475.45, 124.37) |
| NTproBNP | MD | Probiotic yogurt (La5/Bb12) | -12.00 (-89.30, 65.30) |
| NTproBNP | MD | Triple live bacteria | -353.37 (-653.70, -53.03) |
| CRP_hsCRP | SMD | Bifidobacterium quadruple viable | -0.55 (-0.95, -0.14) |
| CRP_hsCRP | SMD | Jinshuangqi | -0.90 (-1.32, -0.49) |
| CRP_hsCRP | SMD | Multi-strain synbiotic | 0.01 (-0.43, 0.45) |
| CRP_hsCRP | SMD | *S. boulardii* | -0.80 (-1.82, 0.22) |
| Readmission | RR | Bifidobacterium triple viable | 0.43 (0.18, 1.03) |
| Readmission | RR | Bifidobacterium viable capsules | 0.41 (0.19, 0.91) |
| Readmission | RR | *S. boulardii* | 0.65 (0.11, 3.75) |
| Mortality | RR | Bifidobacterium triple viable | 3.00 (0.13, 71.91) |
| Mortality | RR | Bifidobacterium viable capsules | 0.25 (0.03, 2.17) |
| Mortality | RR | Multi-strain synbiotic | 0.95 (0.06, 14.69) |
| Mortality | RR | Triple live bacteria | 1.00 (0.06, 15.50) |

*Figure S12.1. Left ventricular ejection fraction (LVEF) detailed formulation-level network map. Edges connect directly compared nodes; edge width and numeral denote the number of direct studies. Saccharomyces boulardii is abbreviated as S. boulardii within the panel. The combined control node includes placebo and usual care.*

*Figure S12.2. Left ventricular ejection fraction (LVEF) detailed formulation-level forest plot versus combined control. Squares are mean differences (MDs) in percentage points, horizontal lines are 95% confidence intervals (CIs), and zero is the null value. Saccharomyces boulardii is abbreviated as S. boulardii within the panel.*

*Figure S12.3. N-terminal pro-B-type natriuretic peptide (NT-proBNP) detailed formulation-level network map. Edges connect directly compared nodes; edge width and numeral denote the number of direct studies. The combined control node includes placebo, usual care, and the non-probiotic food comparator.*

*Figure S12.4. N-terminal pro-B-type natriuretic peptide (NT-proBNP) detailed formulation-level forest plot versus combined control. Squares are mean differences (MDs), horizontal lines are 95% confidence intervals (CIs), and zero is the null value.*

*Figure S12.5. C-reactive protein/high-sensitivity C-reactive protein (CRP/hs-CRP) detailed formulation-level network map. Edges connect directly compared nodes; edge width and numeral denote the number of direct studies. Saccharomyces boulardii is abbreviated as S. boulardii within the panel. The combined control node includes placebo and usual care.*

*Figure S12.6. C-reactive protein/high-sensitivity C-reactive protein (CRP/hs-CRP) detailed formulation-level forest plot versus combined control. Squares are standardized mean differences (SMDs), horizontal lines are 95% confidence intervals (CIs), and zero is the null value. Saccharomyces boulardii is abbreviated as S. boulardii within the panel.*

*Figure S12.7. Readmission detailed formulation-level network map. Edges connect directly compared nodes; edge width and numeral denote the number of direct studies. Saccharomyces boulardii is abbreviated as S. boulardii within the panel. The combined control node includes placebo and usual care.*

*Figure S12.8. Readmission detailed formulation-level forest plot versus combined control. Squares are risk ratios (RRs), horizontal lines are 95% confidence intervals (CIs), and one is the null value. Saccharomyces boulardii is abbreviated as S. boulardii within the panel.*

*Figure S12.9. Mortality detailed formulation-level network map. Edges connect directly compared nodes; edge width and numeral denote the number of direct studies. The combined control node includes placebo and usual care.*

*Figure S12.10. Mortality detailed formulation-level forest plot versus combined control. Squares are risk ratios (RRs), horizontal lines are 95% confidence intervals (CIs), and one is the null value.*

# Appendix 13: Subgroup analyses

Table S13.1. Subgroup-analysis feasibility.

| **Outcome** | **Subgroup variable** | **Subgroup level** | **Analysis class** | **Studies** | **Nodes** | **Direct comparisons** |
| --- | --- | --- | --- | --- | --- | --- |
| LVEF | region | Non-China | pairwise_meta_analysis_only | 2 | 2 | 1 |
| LVEF | region | China | pairwise_meta_analysis_only | 5 | 2 | 1 |
| LVEF | duration_group | duration_ge_12w | network_meta_analysis | 7 | 3 | 2 |
| LVEF | hf_phenotype | Reduced_or_HFrEF | network_meta_analysis | 3 | 3 | 2 |
| LVEF | hf_phenotype | Mixed_or_unspecified | pairwise_meta_analysis_only | 3 | 2 | 1 |
| LVEF | hf_phenotype | HFpEF | not_run | 1 | 2 | 1 |
| NTproBNP | region | Non-China | network_meta_analysis | 2 | 3 | 2 |
| NTproBNP | region | China | pairwise_meta_analysis_only | 3 | 2 | 1 |
| NTproBNP | duration_group | duration_lt_12w | network_meta_analysis | 3 | 4 | 3 |
| NTproBNP | duration_group | duration_ge_12w | pairwise_meta_analysis_only | 2 | 2 | 1 |
| NTproBNP | hf_phenotype | Mixed_or_unspecified | network_meta_analysis | 3 | 3 | 2 |
| NTproBNP | hf_phenotype | Reduced_or_HFrEF | not_run | 1 | 2 | 1 |
| NTproBNP | hf_phenotype | HFpEF | not_run | 1 | 2 | 1 |
| CRP_hsCRP | region | Non-China | network_meta_analysis | 2 | 3 | 2 |
| CRP_hsCRP | region | China | pairwise_meta_analysis_only | 2 | 2 | 1 |
| CRP_hsCRP | duration_group | duration_ge_12w | network_meta_analysis | 2 | 3 | 2 |
| CRP_hsCRP | duration_group | duration_lt_12w | network_meta_analysis | 2 | 3 | 2 |
| CRP_hsCRP | hf_phenotype | Reduced_or_HFrEF | not_run | 1 | 2 | 1 |
| CRP_hsCRP | hf_phenotype | Mixed_or_unspecified | network_meta_analysis | 3 | 3 | 2 |

Table S13.2. Subgroup-analysis estimates versus control.

| **Outcome** | **Subgroup variable** | **Subgroup level** | **Measure** | **Treatment** | **Estimate (95% CI)** |
| --- | --- | --- | --- | --- | --- |
| LVEF | region | Non-China | MD | *S. boulardii* | -0.81 (-3.74, 2.11) |
| LVEF | region | China | MD | Multi-strain bacterial probiotic | 2.14 (-0.14, 4.41) |
| LVEF | duration_group | duration_ge_12w | MD | Multi-strain bacterial probiotic | 2.13 (-0.10, 4.35) |
| LVEF | duration_group | duration_ge_12w | MD | *S. boulardii* | -0.42 (-5.04, 4.20) |
| LVEF | hf_phenotype | Reduced_or_HFrEF | MD | Multi-strain bacterial probiotic | 4.30 (-0.35, 8.95) |
| LVEF | hf_phenotype | Reduced_or_HFrEF | MD | *S. boulardii* | -0.81 (-3.74, 2.11) |
| LVEF | hf_phenotype | Mixed_or_unspecified | MD | Multi-strain bacterial probiotic | 2.32 (-1.41, 6.05) |
| NTproBNP | region | Non-China | MD | Multi-strain synbiotic | -175.54 (-475.45, 124.37) |
| NTproBNP | region | Non-China | MD | Probiotic yogurt (La5/Bb12) | -12.00 (-89.30, 65.30) |
| NTproBNP | region | China | MD | Multi-strain bacterial probiotic | -122.99 (-455.76, 209.77) |
| NTproBNP | duration_group | duration_lt_12w | MD | Multi-strain bacterial probiotic | -231.95 (-492.76, 28.87) |
| NTproBNP | duration_group | duration_lt_12w | MD | Multi-strain synbiotic | -175.54 (-475.45, 124.37) |
| NTproBNP | duration_group | duration_lt_12w | MD | Probiotic yogurt (La5/Bb12) | -12.00 (-89.30, 65.30) |
| NTproBNP | duration_group | duration_ge_12w | MD | Multi-strain bacterial probiotic | -73.50 (-605.75, 458.74) |
| NTproBNP | hf_phenotype | Mixed_or_unspecified | MD | Multi-strain bacterial probiotic | -17.28 (-430.72, 396.16) |
| NTproBNP | hf_phenotype | Mixed_or_unspecified | MD | Multi-strain synbiotic | -175.54 (-783.71, 432.63) |
| CRP_hsCRP | region | Non-China | SMD | Multi-strain synbiotic | 0.01 (-0.43, 0.45) |
| CRP_hsCRP | region | Non-China | SMD | *S. boulardii* | -0.80 (-1.82, 0.22) |
| CRP_hsCRP | region | China | SMD | Multi-strain bacterial probiotic | -0.72 (-1.07, -0.37) |
| CRP_hsCRP | duration_group | duration_ge_12w | SMD | Multi-strain bacterial probiotic | -0.90 (-1.32, -0.49) |
| CRP_hsCRP | duration_group | duration_ge_12w | SMD | *S. boulardii* | -0.80 (-1.82, 0.22) |
| CRP_hsCRP | duration_group | duration_lt_12w | SMD | Multi-strain bacterial probiotic | -0.55 (-0.95, -0.14) |
| CRP_hsCRP | duration_group | duration_lt_12w | SMD | Multi-strain synbiotic | 0.01 (-0.43, 0.45) |
| CRP_hsCRP | hf_phenotype | Mixed_or_unspecified | SMD | Multi-strain bacterial probiotic | -0.72 (-1.07, -0.37) |
| CRP_hsCRP | hf_phenotype | Mixed_or_unspecified | SMD | Multi-strain synbiotic | 0.01 (-0.51, 0.53) |

# Appendix 14: Intervention/comparator and safety clarifications

Table S14.1. Participant accounting and comparator classification. Full-trial, review-eligible-arm, and outcome-specific analysis-set denominators are distinguished.

| **Study** | **Full randomized** | **Eligible arms** | **Comparator** | **Comparator class** | **Analysis status/notes** |
| --- | --- | --- | --- | --- | --- |
| Costanza 2015 | 20 | 20 | Placebo plus standard HF therapy | Placebo | Primary; 8/8 analyzed for LVEF/hsCRP. |
| Awoyemi (GutHeart) 2021 | 151 | 103 | Standard HF care; rifaximin arm excluded | Usual/standard care | Primary; 52/51 eligible randomized; LVEF analysis set 46/45. |
| Karim 2022 | 108 | 108 | Inactive placebo | Placebo | Systematic review only; 53/55 randomized, 44/48 analyzed; no eligible endpoint extracted. |
| Pourrajab 2020/2022 | 90 | 90 | Ordinary yogurt | Active food comparator | Primary where data available; 45/45 randomized, 39/39 analyzed. |
| Matin 2022/2024 | 90 | 90 | Matching placebo | Placebo | Primary; 45/45 randomized, 41/39 analyzed. |
| Ding 2021 | 100 | 100 | Conventional HF therapy | Usual/standard care | Primary; 50/50. |
| Yu 2021 | 10 | 10 | Dietary management | Dietary management | Sensitivity/narrative only; 5/5. |
| Li 2024 | 112 | 112 | ACEI/ARB plus standard HF therapy | Usual/standard care | Primary; 56/56. |
| Chen 2023 | 100 | 100 | Conventional HF treatment | Usual/standard care | Primary; 50/50 for binary outcomes; 44/36 continuous completers. |
| Zhang 2017 | 50 | 50 | Placebo plus standard HF therapy | Placebo | Primary; 25/25. |
| Xu 2024 | 100 | 100 | Glucose-powder placebo plus standard HF therapy | Placebo | Primary; 50/50 randomized, 49/48 analyzed. |
| Zhou 2024 | 90 | 90 | Usual HFpEF therapy | Usual/standard care | Primary; 45/45 randomized; substantial attrition for follow-up assessments. |

**Table S14.2. Detailed intervention characteristics. CFU indicates colony-forming units and NR indicates not reported in the available study report; no species, strain, viable count, adherence, or product-verification detail was inferred from commercial-product knowledge.**

| **Study** | **Intervention/node** | **Species, strain, identifier** | **Dose/viable count** | **Formulation/comparator** | **Duration** | **Adherence assessment** | **Product verification/reporting** |
| --- | --- | --- | --- | --- | --- | --- | --- |
| Costanza 2015 | *S. boulardii* | *S. boulardii*; strain identifier NR | 1000 mg/day; CFU NR | Oral probiotic preparation; placebo + standard HF therapy | 3 months | NR | Manufacturer, batch testing, and viability verification NR. |
| Awoyemi 2021 | *S. boulardii* | *S. boulardii* CNCM I-745 | Two 250-mg capsules twice daily (1000 mg/day); CFU NR | Capsules; standard HF care | 3 months | Returned-tablet count at months 1 and 3; 80% of probiotic arm had >80% compliance. | Fecal *S. boulardii* DNA detected at group level; batch/CFU assay NR. |
| Karim 2022 | Multi-strain bacterial probiotic | Bifidobacteria DSM 24736/24732/24737; lactobacilli DSM 24735/24730/24733/24734; *S. thermophilus* DSM 24731 (species mapping incompletely printed for some IDs) | Vivomixx 112×10^9 CFU once daily | Capsule; inactive placebo | 12 weeks | 9/53 probiotic and 7/55 placebo participants excluded for poor compliance; assessment method NR. | Brand and DSM identifiers reported; independent batch/viability testing NR. |
| Pourrajab 2020/2022 | Probiotic yogurt | *L. acidophilus* La5; *B. lactis* Bb12 | 10^7 CFU/g for both cultures; report states 3×10^9 CFU/day | 300 mL/day low-fat probiotic yogurt; ordinary-yogurt control | 10 weeks | Weekly home delivery; telephone calls and in-person questioning. | PAK Dairy; cold chain and refrigerated storage reported; trial-specific viable-count assay NR. |
| Matin 2022/2024 | Multi-strain synbiotic | *L. casei*, *L. acidophilus*, *L. rhamnosus*, *L. bulgaricus*, *B. breve*, *B. longum*, *S. thermophilus*; strain identifiers NR | Per 0.5-g capsule: 1×10^9, 1.5×10^10, 3.5×10^9, 2.5×10^8, 1×10^10, 5×10^8, and 1.5×10^8 CFU, respectively; FOS 38.5 mg | One capsule/day; matched placebo capsule | 10 weeks | Telephone checks and returned capsule box at final visit. | Zist-takhmir Company; refrigeration reported; independent batch/viability testing NR. |
| Ding 2021 | Multi-strain bacterial probiotic | Jinshuangqi commercial product; species, strain identifiers, and CFU NR in report | 0.5-g tablet; 2 g three times daily (6 g/day) | Tablet plus conventional HF therapy | 12 weeks | NR | Manufacturer and an incompletely printed national product identifier reported; batch/viability verification NR. |
| Yu 2021 | Multi-strain bacterial probiotic | Jinshuangqi commercial product; species, strain identifiers, and CFU NR in report | 0.5-g unit; 2 g three times daily (6 g/day) | Oral product plus dietary management | 6 weeks | NR | Manufacturer and national product identifier S19980004 reported; batch/viability verification NR. |
| Li 2024 | Bifidobacterium viable product | Species and strain identifiers NR | 0.42 g three times daily | Capsule plus ACEI/ARB and standard HF therapy | 4 months | NR | Shanghai Sine; product identifier S10950032; CFU/batch viability NR. |
| Chen 2023 | Bifidobacterium triple viable product | Species and strain identifiers NR | 210 mg/capsule; 630 mg twice daily | Capsule plus conventional HF treatment | 3 months | NR | Shanghai Sine; product identifier S10950032; CFU/batch viability NR. |
| Zhang 2017 | Bifidobacterium viable product | Species, strain identifiers, and CFU NR | 0.42 g three times daily | Capsule; placebo + unchanged standard HF therapy | 4 months | NR | Manufacturer, product identifier, and batch/viability verification NR. |
| Xu 2024 | Bifidobacterium quadruple viable product | *B. infantis*, *L. acidophilus*, *E. faecalis*, *B. cereus*; strain identifiers NR | 0.5-g tablet; 3 tablets three times daily (4.5 g/day); CFU NR | Tablet; glucose-powder placebo + standard HF therapy | 60 days | Active supervision, follow-up, and reminders; quantitative adherence NR. | Product identifier S20060010; 2–8°C dark storage; batch/viability verification NR. |
| Zhou 2024 | *C. butyricum*/enterococcal triple viable product | *C. butyricum*, saccharifying bacterium, *E. faecium*; strain identifiers NR | Per 200-mg tablet: 1×10^5–1×10^8, 1×10^5–1×10^8, and 2×10^5–4×10^8 viable organisms, respectively; 2 tablets three times daily | Tablet plus usual HFpEF therapy | 12 weeks | Daily medication records plus telephone, WeChat, or in-person monitoring; PPS required ≥90% days. | Thesis brand name 适怡; product identifier SJ20160008; storage specified; the English abstract uses inconsistent organism nomenclature; batch viability NR. |

**Table S14.3. Study-specific safety reporting. 'Not reported' is not interpreted as 'no events'.**

| **Study** | **Safety population/definition** | **Intervention-arm events** | **Comparator-arm events** | **Serious events/withdrawals/mortality** | **Reporting limitation** |
| --- | --- | --- | --- | --- | --- |
| Costanza 2015 | Safety denominator unclear | No side effects or adverse events reported | No side effects or adverse events reported | Withdrawals occurred before final 8/8 analysis; attribution detail NR | Absence of reported events cannot establish safety; randomized denominator was 20. |
| Awoyemi 2021 | Eligible randomized arms; adverse events prospectively registered | 5 probiotic participants stopped study medication because of side effects; 2 serious adverse events | 3 serious adverse events in standard-care arm | Eligible-arm serious events adjudicated probably not related (probiotic) or not related (control) | Report inconsistently labels 51/52 between text/flow and Table 1; event definitions differed from other trials. |
| Karim 2022 | NR | NR | NR | 9/53 probiotic and 7/55 placebo participants excluded for poor compliance | No structured adverse-event results identified. |
| Pourrajab 2020/2022 | NR | NR | NR | Mortality/withdrawal by arm not reported as a safety endpoint | No structured adverse-event results identified. |
| Matin 2022/2024 | Analyzed 41 probiotic/39 placebo | Structured adverse-event count NR | Structured adverse-event count NR | Mortality 1/41 vs 1/39 | Mortality was sparse; adverse-event definitions and denominators NR. |
| Ding 2021 | NR | NR | NR | NR | No structured adverse-event results identified. |
| Yu 2021 | HF subgroup 5/5 | NR | NR | NR | Very small subgroup; no structured safety reporting. |
| Li 2024 | Randomized 56/56 | No serious adverse reaction reported; readmission 7/56; mortality 1/56 | No serious adverse reaction reported; readmission 17/56; mortality 4/56 | Serious adverse-reaction counts not itemized | No prespecified AE definition or complete event inventory. |
| Chen 2023 | Randomized 50/50 for clinical events | Readmission 6/50; mortality 1/50 | Readmission 14/50; mortality 0/50 | Structured nonfatal adverse-event reporting NR | Readmission/mortality are clinical outcomes, not a complete safety assessment. |
| Zhang 2017 | NR | NR | NR | NR | No structured adverse-event results identified. |
| Xu 2024 | Completers 49/48; GI symptoms could overlap | Diarrhea 11/49; constipation 14/49; reflux 19/49 | Diarrhea 21/48; constipation 24/48; reflux 23/48 | One control participant withdrew because of severe diarrhea | No single overall AE count or serious-AE framework; symptom categories were not mutually exclusive. |
| Zhou 2024 | Safety set: all 45/45 randomized participants | Any AE 20/45; serious AE 1/45; death 1/45 | Any AE 17/45; serious AE 2/45; death 1/45 | No event judged directly related to study product | Single open-label thesis, short follow-up, and small sample; cannot establish safety. |

Adverse-event definitions, ascertainment, and denominators were too heterogeneous for pooling. The safety evidence was not graded and remains insufficient; absence of demonstrated harm is not interpreted as reassurance.

# Appendix 15: Outcome harmonisation and conversion audit

**Table S15.1. Outcome-level endpoint/change selection and median/IQR conversion audit.**

Endpoint values were used without mixing change scores. Median/IQR conversions used the Wan method on the raw scale and remain approximation-dependent. Ratio-of-means or log-scale synthesis was not feasible because geometric means or consistently transformable summaries were unavailable across contributing studies. Converted-value sensitivities were used only when at least two comparable contrasts remained.

| **Study** | **Outcome** | **Handling** |
| --- | --- | --- |
| Pourrajab 2020 | NT-proBNP | median/IQR to raw-scale mean/SD; approximation-dependent |
| Pourrajab 2022 | sTWEAK | median/IQR to raw-scale mean/SD; approximation-dependent |
| Pourrajab 2022 | sCD163 | median/IQR to raw-scale mean/SD; approximation-dependent |
| Pourrajab 2022 | ADMA | median/IQR to raw-scale mean/SD; approximation-dependent |
| Pourrajab 2022 | LCAT | median/IQR to raw-scale mean/SD; approximation-dependent |
| Pourrajab 2022 | BUN | median/IQR to raw-scale mean/SD; approximation-dependent |
| Chen 2023 | NT-proBNP | median/IQR to raw-scale mean/SD; approximation-dependent |
| Chen 2023 | TMAO | median/IQR to raw-scale mean/SD; approximation-dependent |
| Xu 2024 | NT-proBNP | median/IQR to raw-scale mean/SD; approximation-dependent |
| Xu 2024 | CRP/hs-CRP | median/IQR to raw-scale mean/SD; approximation-dependent |
| Xu 2024 | TNF-alpha | median/IQR to raw-scale mean/SD; approximation-dependent |
| Zhou 2024 | NT-proBNP | median/IQR to raw-scale mean/SD; approximation-dependent |

# Appendix 16: Transitivity and primary network accounting

The LVEF network contains 539 analysis-set participants: combined placebo/usual-care comparator n=265, *S. boulardii* n=54, and bacterial probiotic n=220. It has no active head-to-head comparison and no closed loop. Comparator type, region, HF phenotype, design, duration, and formulation are confounded; this represents serious non-transitivity, so indirect comparisons and P-scores are exploratory.

# Appendix 17: Selected summary of findings and CINeMA

For CINeMA, serious imprecision was assigned if the CI crossed the null or a clinically unimportant range (LVEF ±3 percentage points; NT-proBNP ±100 pg/mL; CRP/hs-CRP SMD ±0.20; RR 0.80–1.25). Serious heterogeneity was assigned when I²≥50% or a prediction interval crossed a decision threshold. Incoherence was not assessable for the star-shaped networks without closed loops. Table S17.1 intentionally presents selected principal comparisons; comparator-stratified TNF-alpha, TMAO, and 6MWT findings remain reported in Table S19.1 and the main text.

**Table S17.1. Selected principal comparisons (not an exhaustive summary of every principal outcome).**

| **Principal comparison** | **Eligible participants** | **Effect estimate** | **Certainty** | **Explicit downgrading reasons** |
| --- | --- | --- | --- | --- |
| LVEF: bacterial probiotic vs usual/standard care | 4 studies; n=382 | REML-HK MD 1.83 (-2.33, 5.98) | Very low | RoB 2, serious heterogeneity, imprecision, comparator restriction |
| LVEF: *S. boulardii* vs control | 2 studies; n=107 | MD -0.42 (-5.04, 4.20) | Very low | RoB 2, very serious imprecision, sparse evidence |
| NT-proBNP: bacterial probiotic vs control | 3 studies; n=265 | MD -122.99 (-455.76, 209.77) | Very low | RoB 2, heterogeneity, imprecision |
| CRP/hs-CRP: bacterial probiotic vs control | 2 studies; n=200 | SMD -0.72 (-1.07, -0.37) | Low | RoB 2, heterogeneity/indirectness, sparse evidence |
| Readmission: bacterial probiotic vs usual/standard care | 2 studies; n=212; 13/106 vs 31/106 events | REML-HK RR 0.42 (0.01, 19.09) | Very low | RoB 2, sparse events, very serious imprecision, selective reporting |
| Mortality: bacterial probiotic vs usual/standard care | 3 studies; n=302; 3/151 vs 5/151 events | REML-HK RR 0.66 (0.03, 14.01) | Very low | RoB 2, sparse events, very serious imprecision |
| Safety: all available study-specific reports | No coherent pooled analysis set; see Table S14.3 | Not pooled | Insufficient; not graded | Sparse and selective reporting, heterogeneous definitions/denominators, and short follow-up; absence of demonstrated harm is not evidence of safety |

# Appendix 18: Post hoc broad-class pairwise analysis

| **Outcome** | **Studies/participants** | **Comparator type(s)** | **Effect vs control (95% CI)** | **I²** | **Note** |
| --- | --- | --- | --- | --- | --- |
| LVEF | k=7; N=539 | standard care/placebo | 1.64 (-0.33, 3.61) | 71.8% |  |
| NT-proBNP | k=5; N=425 | ordinary yogurt; standard care/placebo | -90.66 (-252.24, 70.93) | 64.2% |  |
| CRP/hs-CRP | k=4; N=293 | standard care/placebo | -0.52 (-0.95, -0.08) | 67.0% |  |
| TNF-alpha | k=3; N=309 | standard care/placebo | -2.35 (-3.78, -0.92) | 81.4% |  |
| TMAO | k=3; N=292 | standard care/placebo | -0.07 (-0.10, -0.03) | 69.1% |  |
| Readmission | k=3; N=315 | standard care/placebo | 0.44 (0.25, 0.77) | 0.0% |  |
| Mortality | k=4; N=382 | standard care/placebo | 0.71 (0.19, 2.66) | 0.0% |  |
| Any adverse event | k=1; N=90 | standard care/placebo | 1.18 (0.72, 1.93) | NA | Only one comparison; no pooled estimate. |

This analysis collapses nonexchangeable probiotic and synbiotic interventions and control conditions. It is retained only as a post hoc sensitivity analysis and is not used to infer a uniform class effect.

# Appendix 19: Comparator-stratified robust pairwise sensitivity analyses

Repeated direct comparisons within the same comparator class were synthesized using REML tau-squared estimation and Hartung-Knapp confidence intervals with the IQWiG6 ad hoc correction. These analyses do not mix placebo, usual care, dietary management, or food comparators.

Table S19.1. Comparator-stratified robust pairwise sensitivity analyses.

| **Outcome** | **Comparison** | **k** | **N** | **Effect** | **Estimate (95% CI)** | **I2** |
| --- | --- | --- | --- | --- | --- | --- |
| 6MWT | multi-strain bacterial probiotic vs usual/standard care | 2 | 190 | MD | 13.79 (-0.56, 28.15) | 0.0% |
| LVEF | multi-strain bacterial probiotic vs usual/standard care | 4 | 382 | MD | 1.83 (-2.33, 5.98) | 82.4% |
| Mortality | multi-strain bacterial probiotic vs usual/standard care | 3 | 302 | RR | 0.66 (0.03, 14.01) | 0.0% |
| NT-proBNP | multi-strain bacterial probiotic vs usual/standard care | 2 | 170 | MD | -73.50 (-3524.00, 3377.00) | 87.1% |
| Readmission | multi-strain bacterial probiotic vs usual/standard care | 2 | 212 | RR | 0.42 (0.01, 19.09) | 0.0% |
| TMAO | multi-strain bacterial probiotic vs usual/standard care | 3 | 292 | MD | -0.07 (-0.16, 0.02) | 69.1% |
| TNF-alpha | multi-strain bacterial probiotic vs usual/standard care | 2 | 212 | MD | -3.17 (-5.00, -1.33) | 0.0% |

QoL/ADL was not pooled because ADL, EQ-5D/VAS, and KCCQ measure different constructs. CRP/hs-CRP had no repeated intervention-comparator stratum. NT-proBNP usual-care sensitivity depended entirely on median/IQR conversions and is not interpreted as evidence of effect.

# Appendix 20: Protocol amendments and analysis decisions

Table S20.1 classifies departures from or clarifications to the planned analysis. The PROSPERO record was registered on June 2, 2026 (CRD420261413595). At registration, formal searching/study identification and screening were reported as started, while data extraction, risk-of-bias assessment, and data synthesis had not started. The record listed a review start date of June 3, 2026; the chronology is therefore reported descriptively without an unqualified claim of prospective registration.

| **Item** | **Classification** | **Rationale** | **Effect on interpretation** |
| --- | --- | --- | --- |
| Expanded PubMed, Embase, and Web of Science sensitivity audit | Post hoc search enhancement and update | Test sensitivity to organism, food, and substrate terms without a design filter; update through July 15, 2026 | No additional eligible randomized trial; original PRISMA flow retained and audit reported separately |
| Broad formulation nodes | Analysis amendment | Preserve connected evidence while avoiding a single undifferentiated active class | NMA retained as exploratory |
| Yu 2021 excluded from primary synthesis | Post hoc eligibility/robustness clarification | HF subgroup selection and preservation of randomization could not be confirmed | Study retained only in sensitivity/narrative analyses |
| Comparator-stratified REML Hartung-Knapp analyses | Post hoc robustness analysis | Address nonexchangeable comparators and small k | Wider intervals; conclusions downgraded |
| QoL/ADL pooling removed | Post hoc outcome-harmonisation correction | ADL, EQ-5D/VAS, and KCCQ measure different constructs | Narrative synthesis only |
| Meta-regression and funnel plots removed | Post hoc reporting correction | At most seven and fewer than ten contrasts, respectively | No effect-modification or small-study-effect claims |
| P-scores demoted | Post hoc interpretation change | No closed loops and serious non-transitivity | Descriptive supplement only |
| Outcome-level RoB 2 and explicit CINeMA downgrading | Reporting enhancement | Align certainty with result-specific bias and sparse networks | All synthesized evidence low or very low certainty |
| PROSPERO chronology | Registration-stage clarification | Registered June 2, 2026; searching and screening reported as started; data extraction, risk-of-bias assessment, and data synthesis not started; record-listed review start date June 3, 2026 | Chronology reported descriptively; no unqualified claim of prospective registration |
